# ‘*Candidatus* Pseudomonas auctus’ sp. nov. JDE115 isolated from nodules on soybean (*Glycines max*)

**DOI:** 10.1371/journal.pone.0331920

**Published:** 2025-09-11

**Authors:** Md Sahadat Ali, Fatima Tuz Zohora Mony, Michael Evans, Steven Rideout, David Haak, Paulo Vieira, Jonathan D. Eisenback

**Affiliations:** 1 School of Plant and Environmental Sciences, Virginia Tech, Blacksburg, Virginia, United States of America; 2 Mycology and Nematology Genetic Diversity and Biology Laboratory, United States Department of Agriculture, Agricultural Research Service, Beltsville, Maryland, United States of America; Universidad Autonoma de Chihuahua, MEXICO

## Abstract

A Gram-negative, facultative anaerobic, rod-shaped, motile with peritrichous flagella, fluorescent bacterium, designated ‘*Candidatus* Pseudomonas auctus’ sp. nov. JDE115, was isolated from soybean root nodules in Virginia and characterized using a comprehensive integrative methodology. Growth of JDE115 occurred with 0–5.0% (w/v) NaCl (optimum 1%), at pH 6.0–10.0 (optimum pH 7.0), and at 10–40°C (optimum 28°C) in LB broth. Phylogenetic analyses based on the 16S rRNA gene placed the isolate as a member of a novel species within the genus *Pseudomonas.* Phylogenetic analyses based on whole-genome sequences, 16S rRNA, showed JDE115 having the highest similarity to *Pseudomonas glycinae* MS586. Average Nucleotide Identity (ANI) analysis also revealed the highest similarity of JDE115 to *Pseudomonas glycinae* MS586 (94.59%), which is below the 95% threshold for species delineation. Genome-to-genome distance analysis (GGDC, Formula 2) showed a maximum value of 57.10% with the same strain, far below the 70% cutoff. The primary isoprenoid quinone detected in JDE115 was ubiquinone-9 (Q-9) and the DNA G + C content was 60.68 mol%. The whole-cell fatty acid profile was dominated by C16:0, C17:0 cyclo, and the summed features 3 (C16:1ω7c and/or C16:1ω6c) and 8 (C18:1ω7c and/or C18:1ω6c). Additional fatty acids detected included 12:0, 14:0, and 18:0. Based on these phenotypic, chemotaxonomic, and phylogenetic data, strain JDE115 is proposed to represent a new species in the genus *Pseudomonas*, for which the name ‘*Candidatus* Pseudomonas auctus’ sp. nov. is proposed.

## Introduction

The genus *Pseudomonas* was first proposed by Migula in 1894 and is known for its remarkable versatility, metabolic diversity, and environmental ubiquity [1,2]. Members of this genus are Gram-negative [3,4], rod-shaped bacteria [5] that are non-spore-forming [6] and motile due to polar flagella [6,7]. They are catalase [8,9] and oxidase-positive [10], and their ability to thrive in diverse habitats, including plants, soil, animals, and water, highlights their adaptability and ecological significance [11–16]. Currently, more than 200 species of *Pseudomonas* are listed in the bacterial names withstanding in nomenclature (https://www.bacterio.net). Many species within this genus exhibit plant growth-promoting [17–20] and biocontrol [3,4,14,17,21] properties, making them vital in agriculture for sustainable crop production and disease management. These bacteria contribute to plant health through mechanisms such as phosphate solubilization [22–27], siderophore production [28–32], and the suppression of plant pathogens via direct antagonism or secondary metabolite production [33–36].

However, accurate identification and classification of bacterial species, especially within the diverse genus *Pseudomonas*, require a multifaceted approach [37–40]. Among the most robust methods for delineating new species are genomic and phylogenetic analyses [37,41–43]. Average Nucleotide Identity (ANI) is widely used for genomic comparisons, with a threshold of 95% often considered indicative of species delineation [44–47]. Similarly, digital DNA-DNA Hybridization (dDDH) [48–49], which provides an in silico estimate of DNA-DNA reassociation, has a 70% cutoff for species-level distinctions [48,50]. These genome-based metrics complement traditional phylogenetic analyses, such as those based on the 16S rRNA gene, which has been a gold standard for bacterial taxonomy [51–53], and provide additional resolution for closely related taxa [54–58].

Chemotaxonomic approaches further strengthen taxonomic characterization by analyzing cellular fatty acids, respiratory quinones, and polar lipid profiles [59–61]. Fatty Acid Methyl Ester (FAME) analysis serves as a complementary or alternative method for detecting soil organisms. It is widely used to characterize microbial community structures and as a diagnostic tool for identifying specific organisms in environmental samples [62]. FAME analysis has also been instrumental in differentiating species within *Pseudomonas* [63–66]. Specific FAMEs or their ratios can indicate the presence and abundance of certain taxa, serving as biomarkers. For example, the presence of 10:0 3OH, 12:0 3OH, and 14:0 3OH fatty acids indicates a gram-negative organism, while their absence, along with LPS, suggests a gram-positive organism [67]. Respiratory quinones, such as ubiquinone Q-9, and polar lipids, including phosphatidylethanolamine and phosphatidylglycerol, also provide distinctive chemotaxonomic markers [68–70].

Phenotypic and physiological traits play a critical role in the identification and classification of *Pseudomonas* species [71,72]. These bacteria are characterized by their Gram-negative cell wall structure [4,19,73], essential for differentiating them from Gram-positive taxa. Their rod-shaped morphology and the presence of one or more polar flagella confer motility, enabling them to colonize diverse environments efficiently [74–76]. Non-spore-forming and catalase-positive traits are hallmark features that further delineate members of *Pseudomonas* from other genera [40]. Additionally, their oxidase-positive nature reflects their reliance on aerobic respiration, a trait that supports their adaptability in various habitats [10]. These phenotypic traits not only confirm their inclusion within *Pseudomonas* but also provide initial clues for distinguishing closely related species. By integrating these phenotypic and physiological attributes with genomic, phylogenetic, and chemotaxonomic analyses, a comprehensive framework for species identification is achieved. Thus, these comprehensive polyphasic approaches are instrumental in accurately determining the taxonomic position of ‘*Candidatus* Pseudomonas auctus’ sp. nov. JDE115.

## Materials and methods

### Organism and growth condition

*‘Candidatus* P. auctus’ sp. nov. JDE115 was isolated from surface-sterilized root nodules of soybean (*Glycine max* (L.) Merr.). Briefly, 0.25 g of surface-sterilized soybean nodules (P1) were homogenized and suspended in 0.9% NaCl solution. The suspension was plated onto King’s B medium for bacterial isolation (P2). A single colony of the strain was cultured in LB (Luria–Bertani) lite broth at 28°C for 24 hours under continuous shaking at 200 rpm (P3). Immediately, five samples for glycerol stock (40%) were prepared and stored at −80°C (P4).

### Cell morphology and physiological tests

The morphological characteristics of strain JDE115 colonies were assessed following growth on LB lite agar plates. Gram staining was performed according to the protocol described by Bartholomew & Mittwer [77]. Cell morphology and flagellation types were examined using 120 kV FEI T12 Tecnai Spirit transmission electron microscope (TEM) with routine negative staining using Urany-Less^®^ [78] (P5). The attachment of cells to the root surface was observed using a Jeol NeoScope^®^ JCM 5000 scanning electron microscope (SEM) operating at 10kV after sputter coating with 90Å of gold using an Emitech^®^ SC7620 mini sputter coater [79,80]. Motility was visualized and recorded using a Leitz^®^ Dialux 22 bright field and phase contrast light microscope equipped with a Nikon^®^ D300 camera to create photomicrographs and digital video [81]. Fluorescent pigment production was evaluated by growing JDE115 on King’s B medium at 28°C for 24 hours. After incubation, fluorescence was examined using a UV transilluminator (Avantor) under long-wave UV light (365 nm, UV-A) in a darkened environment. Plates were placed directly on the transilluminator surface for visualization [82]. Optimal growth conditions for JDE115 were determined by measuring optical density (OD600) in LB liquid cultures [83,84]. Growth was evaluated at temperatures ranging from 4°C to 40°C in 4°C increments over 24 hours and at pH levels from 4.0 to 10.0 to determine the strain’s tolerance and preferences [2,73,85].

Physiological assessments, including salt tolerance, pH range, temperature range, motility, and aerobic metabolism, alongside comprehensive biochemical tests (unpublished, Ali et al.), were conducted to characterize ‘*Candidatus* Pseudomonas auctus’ nov. sp. JDE115. Cellular fatty acid profiles were analyzed using the Sherlock 6.1 system (Microbial Identification Inc.) with the RTSBA6 library [67], providing detailed insights into the strain’s fatty acids composition (P6). Biochemical properties and enzymatic activities were evaluated using Biolog^®^ GENIII Microplates [86–89] (P7). Results were recorded after 48 hours of incubation at 28°C, yielding a robust dataset highlighting the metabolic versatility and adaptive features of JDE115.

### Phylogenetic analysis

Bacteria from glycerol stock were streaked onto Luria-Bertani agar (LBA) plates and incubated to obtain isolated colonies. A single colony was transferred into 50 mL of LB lite broth (containing 5 g/L yeast extract and 10 g/L NaCl) and incubated at 28°C for 16 hours with continuous shaking at 200 rpm. The culture reached an optical density (OD) of approximately 0.6. From this actively growing culture, 1.8 mL was used for genomic DNA extraction using the DNeasy UltraClean Microbial Kit (Qiagen^®^, USA) (P8). The extracted DNA was quantified using a NanoDrop 1000 spectrophotometer and a Qubit 3.0 fluorometer (Thermo Fisher Scientific^®^, USA). DNA purity was further assessed by agarose gel electrophoresis (P9). A nearly full-length 16S rRNA gene was amplified using the universal primers 27F (5′-AGAGTTTGATCMTGGCTCAG-3′) and 1492R

(5′-TACGGHTACCTTGTTACGACTT-3′) through PCR [73,90,91]. PCR was performed using a Bio-Rad^®^ Thermal Cycler, and the products were purified with a QIAquick^®^ PCR Purification Kit (QIAGEN^®^) (P10). Sanger sequencing reactions were carried out by the ABI 3730XL.

Computation of the phylogenetic tree based on 16S rRNA was performed using SeaView^®^ [92,93] with the maximum likelihood approach with the 1000 bootstrap replicates [94,95]. Sequences of type strains used in the phylogenetic analysis were downloaded from NCBI (accession numbers in Table 3).

The genomic DNA was sheared into fragments using a Covaris^®^ S220 ultrasonicator, and sequencing libraries were prepared using the Illumina-compatible NEBNext Ultra II DNA Library Prep Kit (New England BioLabs^®^, USA). Sequencing was performed on the Illumina^®^ NovaSeq PE150 platform at the NGS core facility, generating paired-end reads of 150 bp. FASTQ files were generated using Guppy (v4.4.1), and sequences with a Phred score below 7 were excluded. De novo assembly was performed using Flye (v2.9) [96], with default parameters and a genome size setting of 6.2 Mbp. Assembly completeness was confirmed using dot plots generated with Gepard [97]. The complete genome was visualized using CGView [98,99]. Genome annotation was conducted using the NCBI Prokaryotic Genome Annotation Pipeline (PGAP) and the RAST server [100]. The Whole Genome Shotgun sequence project has been deposited in GenBank under accession number PRJNA1196930.

Similarity analyses (ANI and GGDC) of the sequenced genome of strain JDE115 to other 40 genomes of the closely related *Pseudomonas* species were determined as briefed below. ANI based on pairwise comparison was calculated using the software FastANI [44]. GGDC was calculated using the web service http://ggdc.dsmz.de and using the recommended BLAST+method [49]. The GGDC results shown are based on the recommended formula 2 (sum of all identities found in HSPs divided by the overall HSP length), which is independent of the genome length and is thus robust against the use of incomplete draft genomes.

The Type (Strain) Genome Server (TYGS, https://tygs.dsmz.de) was employed for whole-genome-based taxonomic analysis, incorporating recent methodological updates [101,102]. TYGS identified closely related type strains through two complementary approaches: (1) comparison of user genomes with type strain genomes in the TYGS database using the MASH algorithm [103] to identify the ten closest type strains and (2) extraction of 16S rDNA sequences from user genomes using RNAmmer [104], followed by BLAST [105] analysis against 16S rDNA gene sequences from 22,195 type strains in the TYGS database. These methods were used to determine the closest type strain genomes based on intergenomic distances calculated using the Genome Blast Distance Phylogeny (GBDP) approach with the “coverage” algorithm and distance formula d_5_ [49]. Pairwise genome comparisons were performed with GBDP to infer intergenomic distances and calculate digital DNA-DNA hybridization (dDDH) values using GGDC 4.0. Phylogenomic analysis was conducted by constructing a balanced minimum evolution tree using FASTME 2.1.6.1 with 100 pseudo-bootstrap replicates for branch support [106]. The tree was rooted at the midpoint [107] and visualized using PhyD3 [108]. Species clustering was based on a 70% dDDH threshold [102], while subspecies clustering used a 79% dDDH threshold, as previously described [109]. These analyses provided insights into the taxonomic positioning of the studied genome. Taxonomic and nomenclatural information was also supplemented by the List of Prokaryotic Names with Standing in Nomenclature (LPSN, https://lpsn.dsmz.de) [101].

### Chemotaxonomic analysis

The cellular fatty acid profile of JDE115, a critical chemical characteristic for bacterial identification, was analyzed to support its taxonomic classification. Fatty acids were harvested after 24 hours of incubation at 28°C on Tryptic Soy Agar (TSA). The extraction process involved saponification, methylation, and subsequent extraction following the standardized protocols of the MIDI System (Sherlock Microbial Identification System, version 6.0B). Analysis was performed using gas chromatography (GC; 6850, Agilent Technologies^®^), and the fatty acids were identified utilizing the TSBA6.0 database of the Microbial Identification System [67].

## Results and discussion

### Phenotype analysis

**Fig 1:**
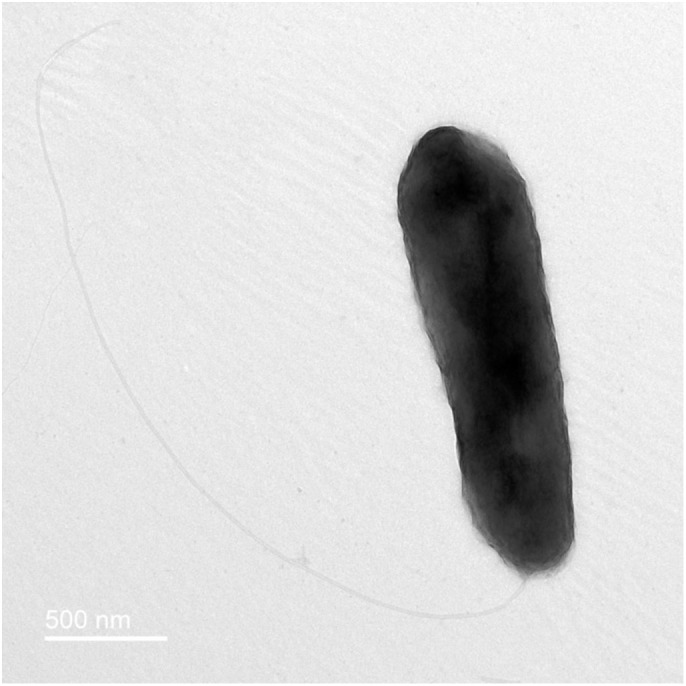
Transmission electron micrograph of ‘*Candidatus* Pseudomonas auctus’ nov. sp. JDE115. This rod-shaped bacterium has a monotrichous polar flagellum that is more than twice as long as its body.

‘*Candidatus* P. auctus’ sp. nov. JDE115 was observed to be gram-negative, rod-shaped (0.5 µm) (Fig 1), fluorescent (S1 Fig), aerobic or facultatively anaerobic, motile (S1 Movie) with monotrichous flagella (Fig 1). Colonies grown on LB at 28°C for 24 h are light yellow, round, smooth, convex, waxy and translucent, 4.0–5.0 mm in diameter (S2 Fig). Growth occurs in 0–4% (w/v) NaCl (optimum 1%), at pH 4.0–10.0 (optimum pH 7.0), and at 10–40°C (optimum 28 °C). Fluorescent pigments were observed when cultured for 24 hr at 28°C on King’s B medium (S1 Fig). The physiological, morphological, and phenotypic characteristics in Biolog GEN III tests, which showed differentiation of strains JDE115 from other closely related *Pseudomonas* species, are listed in Table 1.

**Table 1:** Differentiating characteristics of ‘*Candidatus* Pseudomonas auctus’ sp. nov. strain JDE115 from other related species of *Pseudomonas.*

Characteristics	*P. auctus* JDE115	*P. glycinae* MS586^T^	*P*. *kribbensis* 46-2^T^	*P*. *granadensis* F-278	*P*. *moraviensis* 1B4^T^	*P*. *koreensis* Ps9-14^T^	*P*.*baetica* a390T	*P*. *vancouverensis* DhA-51^T^	*P*. *jessenii* DSM 17150^T^	*P*. *reinekei* MT1^T^
Flagellation	Polar, Single	Polar, multiple	Polar, multiple	Polar, two	Polar, two	Polar, multiple	ND	ND	Polar, single	ND
Fluorescence										
Growth at 4°C								ND^a^		ND
Tolerance of NaCl at 5%										
Nitrate reduction										
Arginine dihydrolase										
Hydrolysis of gelatin										
Citrate utilization										
Urease						ND				
Assimilation of l-Arabinose										
N-Acetyl-d-glucosamine										
Phenylacetic acid										
d-Mannose										
Dextrin										
Tween-40										
d-Cellobiose										
d-Trehalose										
l-Arabinose										
d-Fructose								ND		
d-Mannitol										
d-Arabitol										
l-Alanine										
l-Serine										
α-Ketobutyric acid										
α-Ketoglutaric acid										
Glucuronamide										
l-Histidine										
d-Serine										
d-Galactose										
d-Galacturonic acid			ND			ND				
d-Glucuronic acid										
Glucuronamide					ND					
p-Hydroxy phenylacetic acid										
Quinic acid										
d-Saccharic acid										
Glycyl-l-proline			ND							
L-Pyroglutamic acid						ND				
Inosine										
Propionic acid										
Formic acid										
Acetic acid										
Methyl pyruvate										
GC content (%)	60.7	60.5	60.5	59.9	60.3	59.1	58.7	67.2	62.2	59.1

Comparison of key phenotypic and biochemical characteristics of ‘*Candidatus* Pseudomonas auctus’ nov. sp. JDE115 with closely related *Pseudomonas* species, including flagellation type, fluorescence, growth at 4°C, NaCl tolerance, nitrate reduction, and various metabolic and enzymatic activities. Biochemical reactions were assessed using the Biolog GEN III system, which evaluates the utilization of 71 carbon sources and the resistance to 23 chemical agents for comprehensive bacterial identification. Data for strain ‘*Candidatus* Pseudomonas auctus’ nov. sp. JDE115 were obtained in this study. Data for other type strains were obtained from references, *P. glycinae* MS586^T^ Jia et al. 2020 [73]; *P*. *kribbensis* 46-2^T^ Chang et al. 2016 [110]; *P*. *granadensis* F-278,770^T^ Pascual et al. 2015 [111]; *P*. *moraviensis* 1B4^T^ Pascual et al. 2015 [111]; *P*. *koreensis* Ps9-14^T^ Tvrzová et al. 2006 [112]; *P*.*baetica* a390^T^ López et al. 2012 [113]; *P*. *vancouverensis* DhA-51^T^ Cámara et al. 2007 [114]; *P*. *jessenii* DSM 17150^T^ Cámara et al. 2007 [114]; *P*. *reinekei* MT1^T^ Cámara et al. 2007 [114].

Abbreviations: dark green is for positive reaction; red is for negative reaction; light green is for weak reaction; and ND^a^ is for no data.

### Phylogenetic analysis

Sequence analysis of the 16S rRNA gene revealed that ‘*Candidatus* P. auctus’ nov. sp. JDE115 shares significant sequence identity (>99%) with several *Pseudomonas* species. The closely related strains include *P. glycinae* MS586^T^ (99.86%), *P. kribbensis* 46-2^T^ (99.71%), *P. soyarea* JJL17^T^ (99.36%), *P. koreensis* Ps 9-14^T^ (99.36%), *P. reinekei* MT1^T^ (99.29%), and *P. moraviensis* 1B4^T^ (99.29%). Due to the high sequence similarity, the 16S rRNA gene alone cannot determine the precise taxonomic position of these closely related species [115,116]. This limitation arises from the high conservation of 16S rRNA variable regions, taxon-specific evolutionary constraints, and detection biases, which often hinder its ability to resolve within-genus taxonomy accurately [117]. Recent studies have also highlighted that evolutionary rigidity and horizontal gene transfer associated with the 16S rRNA gene contribute to insufficient diversification among closely related taxa, resulting in limited resolution at the species level [118]. Further whole-genome analyses and phenotypic characterizations are required to clarify the taxonomic placement and distinguish ‘*Candidatus* P. auctus’ nov. sp*.* JDE115 from its closely related species.

**Fig 2:**
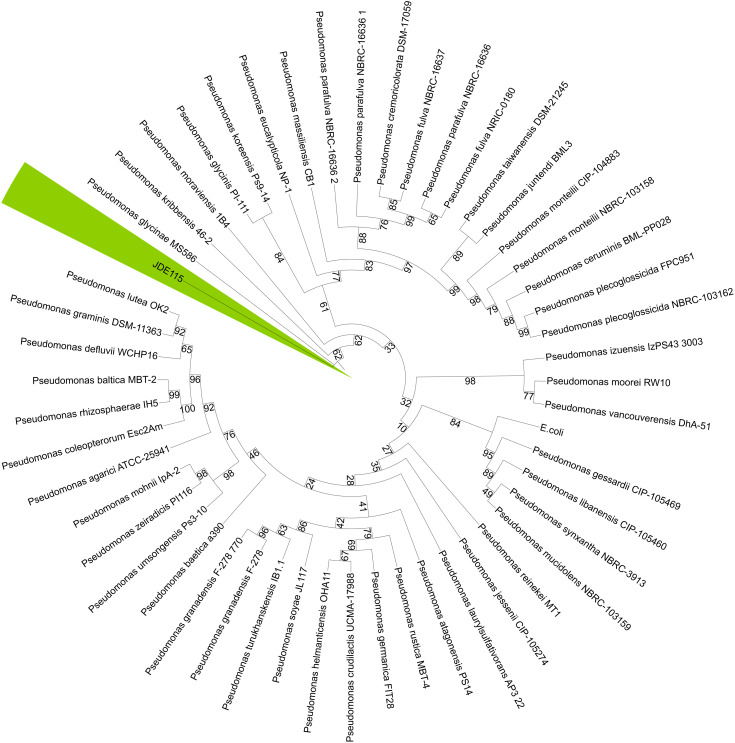
Phylogenetic tree illustrating the evolutionary relationship of ‘*Candidatus* Pseudomonas auctus’ nov. sp. JDE115 with closely related species based on 16S rRNA gene sequences. The tree was created using the Maximum Likelihood method through the PhyML engine integrated into SeaView software [92]. Bootstrap values above 50% are shown at the branch nodes, supporting the branching patterns. This analysis highlights the high sequence similarity between JDE115 and other species within the genus *Pseudomonas* while demonstrating the distinct taxonomic position of JDE115 through a unique branch with significant bootstrap support (highlighted in green).

However, A phylogenetic tree based on the 16S rRNA gene sequences was constructed to assess the evolutionary relationship of ‘*Candidatus* P. auctus’ nov. sp*.* JDE115 with other closely related species. The analysis revealed that JDE115 shares a high sequence similarity with multiple *Pseudomonas* species, including *P. glycinae* MS586^T^, *P. kribbensis* 46-2^T^, and *P. soyarea* JJL17^T^, forming a distinct cluster within the *Pseudomonas* genus. Despite the close relationships, JDE115 formed a unique branch with high bootstrap support, indicating its distinct taxonomic position (Fig 2).

### General genome features

The genome of ‘*Candidatus* Pseudomonas auctus’ nov. sp*.* JDE115 was fully sequenced and annotated, revealing a genome size of 6,183,199 base pairs (bp) with a GC content of 60.68%, the genome size of *Pseudomonas* typically range from 4.2 to 7Mba [119,120]. Besides, the GC content of JDE115 falls within the reported range of 48–68 mol% for members of the genus *Pseudomonas* [121–125]. The genome comprises 5,648 genes, of which 5,509 (97.54%) are coding genes and 70 (1.24%) are RNA genes. Additionally, the genome contains 69 pseudogenes (1.22%), representing a minor fraction of the total genetic content. The coding DNA sequence (CDS) accounted for 5,578 genes, contributing to 98.76% of total genes (Table 2). The number of coding sequences (CDS) within the genus of *Pseudomonas* exhibits substantial variability, ranging from 4,274–6,305 [126].

**Table 2:** General taxonomic genome features of ‘*Candidatus* Pseudomonas auctus’ nov. sp*.* JDE115.

Attributes	Value	% of total
Genome size (bp)	6,183,199	100
DNA Coding (bp)	5,513,388	89.17
GC Content (%)	3,752,162	60.68
Genes (total)	5,648	100
CDS (total)	5,578	98.76
Genes (coding)	5,509	97.54
Genes (RNA)	70	1.24
Pseudo Genes	69	1.22

Summary of the genome assembly and annotation of ‘*Candidatus* Pseudomonas auctus’ nov. sp*.* JDE115, including genome size, GC content, total gene count, coding sequences (CDS), RNA genes, and pseudogenes.

The ANI analysis revealed the highest similarity of ‘*Candidatus* P. auctus’ nov. sp*.* JDE115 with *P. glycinae* MS586, with a value of 94.59%, followed by *P. fitomatiaceae* FIT81 (94.24%) and *P. guzikowskii* IzPS32d (93.92%). However, these values fall below the commonly accepted ANI threshold of 95% for species delineation [44]. ANI comparisons between genomes and type strain assemblies have corrected approximately 750 previously misidentified entries in GenBank, underscoring its critical role in taxonomic accuracy [127]. Additionally, ANI’s ability to handle large datasets with high computational efficiency makes it ideal for modern microbial taxonomy, supporting robust species-level classifications even in complex datasets [128]. Similarly, digital DNA–DNA hybridization (dDDH, calculated using GGDC) values ranged from 57.10% with *P. glycinae* MS586 to as low as 21.30% (*P. wenzhouensis* A20) with more distantly related species (Table 3).

**Table 3:** ANI (%) and dDDH (%) between ‘*Candidatus* Pseudomonas auctus’ nov. sp. JDE115 and closely related sequenced strains of the genus *Pseudomonas.*

*Pseudomonas* species	Genome accession number	ANI (%)	DDH (%)
*P. glycinae* MS586	GCF_001594225.2	94.59	57.10
*P. fitomaticsae* FIT81	GCF_021018765.1	94.24	54.80
*P. gozinkensis* IzPS32d	GCF_014863585.1	93.92	54.00
*P. allokribbensis* IzPS23	GCF_014863605.1	91.74	43.00
*P. kribbensis* 46−2	GCF_003352185.1	91.46	42.50
*P. monsensis* PGSB 8459	GCF_014268495.2	88.43	33.60
*P. zeae* OE 48.2	GCF_014268485.2	88.20	33.00
*P. tensinigenes* ZA 5.3	GCF_014268445.2	88.12	32.90
*P. germanica* FIT28	GCF_019614655.1	88.07	32.70
*P. iranensis* SWRI54	GCF_014268585.2	87.67	32.10
*P. hamedanensis* SWRI165	GCF_014268595.2	87.32	31.70
*P. grandcourensis* DGS24	GCF_039909015.1	86.53	29.60
*P. hormoni* G20-18	GCF_018502625.1	86.41	29.60
*P. azerbaijanorientalis* SWRI123	GCF_019139795.1	86.41	29.90
*P. nunensis* ln5	GCF_024296925.1	86.29	29.30
*P. wuhanensis* FP607	GCF_030687395.1	86.15	29.30
*P. svalbardensis* PMCC 200367	GCF_030053115.1	86.10	29.00
*P. cucumis* FP1935	GCF_030687935.1	86.07	28.90
*P. silesiensis*	GCF_001661075.1	85.76	29.30
*P. purpurea* DGS26	GCF_039908635.1	85.30	27.20
*P. chlororaphis* ATCC 9446	GCF_036689615.1	85.12	26.80
*P. helvetica* DGS28	GCF_039908645.1	85.01	27.00
*P. ogarae* SWRI108	GCF_014268695.2	84.60	26.90
*P. viciae* 11K1	GCF_004786035.1	84.53	26.50
*P. alvandae* SWRI17	GCF_019141525.1	84.29	26.60
*P. protegens* CHA0	GCF_900560965.1	84.16	25.40
*P. hefeiensis* FP205	GCF_030687835.1	84.03	26.10
*P. shahriarae* SWRI52	GCF_014268455.2	83.69	25.10
*P. pergaminensis* 1008	GCF_024112395.2	83.64	24.90
*P. asgharzadehiana* SWRI132	GCF_019139815.1	83.53	24.60
*P. tritici* SWRI145	GCF_014268275.3	83.42	24.50
*P. salmasensis* SWRI126	GCF_014268375.2	83.33	24.80
*P. vanderleydeniana* RW8P3	GCF_014268755.2	83.30	24.80
*P. sessilinigenes* CMR12a	GCF_003850565.1	83.07	24.30
*P. mucidolens* NCTC8068	GCF_900475945.1	82.91	24.40
*P. tructae* SNU WT1	GCF_004214895.1	82.11	22.90
*P. abieticivorans* PIA16	GCF_023509015.1	81.89	23.10
*P. oryziphila* 1257	GCF_003940825.1	81.76	22.70
*P. muyukensis* COW39	GCF_019139535.1	81.73	22.40
*P. eucalypticola* NP-1	GCF_013374995.1	81.63	22.80
*P. fakonensis* COW40	GCF_019139895.1	81.63	22.40
*P. versuta* L10.10	GCF_001294575.1	81.62	23.30
*P. maumuensis* COW77	GCF_019139675.1	81.58	22.10
*P. xantholysinigenes* RW9S1A	GCF_014268885.2	81.56	22.40
*P. xanthosomatis* COR54	GCF_019139835.1	81.56	22.60
*P. entomophila* L48	GCF_000026105.1	81.52	22.40
*P. lijiangensis* LJ2	GCF_018968705.1	81.44	22.60
*P. taetrolens* NCTC10697	GCF_900475285.1	81.34	23.10
*P. cichorii* DSM 50259	GCF_018343775.1	81.31	22.60
*P. anuradhapurensis* RD8MR3	GCF_014269225.2	81.24	22.70
*P. rhizosphaerae* DSM 16299	GCF_000761155.1	81.24	22.80
*P. putida* NBRC 14164	GCF_000412675.1	81.24	22.20
*P. fortuita* GMI12077	GCF_026898135.2	81.05	22.10
*P. syringae* pv. tomato str. DC3000	GCF_000007805.1	80.87	22.50
*P. cavernae* K2W31S-8	GCF_003595175.1	80.72	21.90
*P. huanghezhanensis* BSw22131	GCF_026810445.1	80.40	22.00
*P. promysalinigenes* RW10S1	GCF_014269025.2	80.36	21.60
*P. syringae* pv. *Tagetis* ICMP 4091	GCF_022557255.1	80.32	22.00
*P.* *tohonis* TUM18999	GCF_012767755.2	80.18	21.30
*P. wenzhouensis* A20	GCF_021029445.1	80.10	21.60

The comparative genomic analysis underscored the unique genetic identity of ‘*Candidatus* P. auctus’ nov. sp*.* JDE115. While ANI and dDDH analyses identified significant genomic similarities with certain *Pseudomonas* species, the values consistently remained below the established cutoffs (95% for ANI and 70% for dDDH) for species-level classification [129]. Notably, JDE115 shared substantial genomic relatedness with species like *P. glycinae* MS586 (94.59 and 57.10), *P. fitomaticsae* FIT81 (94.24 and 54.80), *P. gozinkensis* IzPS32d (93.92 and 54.00), *P. kribbensis* 46−2 (91.46% ANI and 42.50% dDDH) and *P. alokibbiensis* IzPS23 (91.74% ANI and 43.00% dDDH), yet the data unequivocally supports the novelty of JDE115 as a distinct taxonomic entity.

Average Nucleotide Identity (ANI) and dDDH values were calculated using the Genome-to-Genome Distance Calculator (GGDC). ANI values above 95% typically indicate species-level relatedness, while dDDH values above 70% suggest the same species.

**Fig 3:**
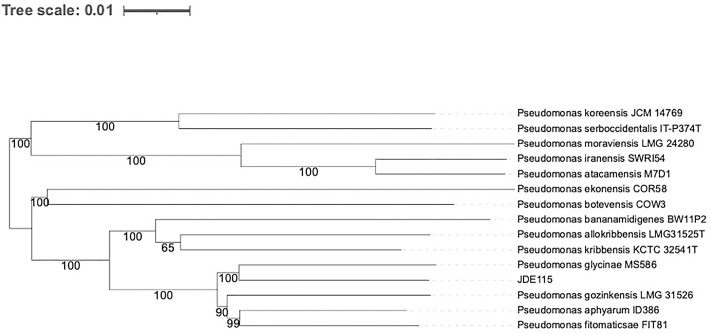
Whole-genome sequence tree generated with TYGS for ‘*Candidatus* Pseudomonas auctus’ nov. sp. JDE115 and its closely related species of the genus *Pseudomonas.* Tree inferred with FastME from GBDP distances was calculated from genome sequences. Branch lengths are scaled in terms of GBDP distance formula d5; numbers above branches are GBDP pseudo-bootstrap support values from 100 replications. Accession numbers of sequences used in this study are summarized in Table 4.

A whole-genome sequence-based phylogenetic analysis was conducted for ‘*Candidatus* P. auctus’ nov. sp*.* JDE115 and closely related species within the genus *Pseudomonas*. The tree was inferred using the Genome BLAST Distance Phylogeny (GBDP) approach implemented in the TYGS platform. Distances were calculated from genome sequences using formula d5. The resulting phylogenetic tree illustrated that JDE115 formed a distinct branch within the genus, clustering most closely with *P. glycinae* MS586 and *P. kribbensis* KCTC 32541^T^. Branch lengths in the tree are scaled in terms of GBDP distance formula d5, and bootstrap values from 100 replicates are provided at each node, indicating strong statistical support for the inferred relationships. High bootstrap support values (≥90) were observed, particularly in the branching patterns distinguishing ‘*Candidatus* P. auctus’ nov. sp*.* JDE115 from its closest relatives, reinforcing its phylogenetic distinctiveness (Fig 3). The closest relatives of JDE115 in the phylogenetic tree include *P. glycinae* MS586 (TYGS ID: 37260), *P. kribbensis* KCTC 32541^T^ (TYGS ID: 14671), and *P. moraviensis* LMG 24280 (TYGS ID: 19005). The accession numbers and strain names of all related species are summarized in Table 4. These data underscore the unique taxonomic position of JDE115 within the genus *Pseudomonas* based on whole-genome analysis. Having established the genomic distinctiveness of JDE115, its chemotaxonomic characteristics were studied to further validate its taxonomic classification.

**Table 4:** Accession numbers for genome sequences of various *Pseudomonas* species strains utilized in whole-genome phylogenetic analysis.

Species	Biosample accession	Strains name	TYGS ID	Isolation Source	Authority
*P. bananamidigenes*	SAMN04444410	BW11P2	100258	Rhizosphere	[122]
*P. aphyarum*	SAMN28093775	ID386	104585	Aquatic environments	[124]
*P. fitomaticsae*	SAMN19241968	FIT81	109195	Rhizosphere	[121]
*P. kribbensis*	SAMN09244908	32541^T^	14671	Soil or environmental	[110]
*P. iranensis*	SAMN15248341	SWRI54	147537	Rhizosphere	[122]
*P. ekonensis*	SAMN19473669	COR58	147556	Rhizosphere	[122]
*P. moraviensis*	SAMN04490196	LMG 24280	19005	**Unknown**	[112]
*P. atacamensis*	SAMN11356701	M7D1	22501	Rhizosphere	[116]
*P. glycinae*	SAMN04435621	MS586	37260	Rhizosphere	[73]
*P. koreensis*	SAMD00245249	JCM 14769	48496	Soil or environmental	[130]
*P. botevensis*	SAMN19473668	COW3	82604	Rhizosphere	[122]
*P. serboccidentalis*	SAMN29793685	IT-P374^T^	86200	Rhizosphere	[125]
*P. gozinkensis*	SAMN16250066	LMG 31526	92631	Soil or environmental	[131]
*P. allokribbensis*	SAMN16250065	LMG31525^T^	92633	Soil or environmental	[131]
‘*Candidatus* Pseudomonas auctus’ nov. sp.	SAMN45708326	JDE115	U884796	Rhizosphere	This study

This table provides the genome accession numbers, strain names, TYGS ID, and isolation sources of *Pseudomonas* species used in the whole-genome sequence-based phylogenetic analysis. ‘*Candidatus* Pseudomonas auctus*’* nov. sp. JDE115 was sequenced in this study.

### Chemotaxonomic analysis

Cellular fatty acids were analyzed for ‘*Candidatus* P. auctus’ nov. sp*.* JDE115 using the Sherlock 6.1 system (Microbial Identification Inc.) and the RTSBA6 library [67]. The dominant fatty acids detected for JDE115 included C16:0 (25.07%), summed feature 3 (C16:1ω7c/C16:1ω6c) (34.78%), summed feature 8 (C18:1ω7c/C18:1ω6c) (16.91%), and C17:0 cyclo (0.87%). Additionally, fatty acids C12:0 (1.88%), C14:0 (0.66%), and C18:0 (1.35%) was identified (Table 5). These profiles are consistent with those typically observed in the genus *Pseudomonas*. The presence of three characteristic fatty acids—C10:0 3-OH, C12:0, and C12:0 3-OH—further confirms the taxonomic affiliation of JDE115 within *Pseudomonas*.

**Table 5:** Distinct cellular fatty acid profiles of strain ‘*Candidatus* Pseudomonas auctus’ nov. sp. JDE115 compared with related *Pseudomonas* strains.

Fatty acid	*P. auctus* JDE115	*P. glycinae* MS586^T^	*P*. *kribbensis* 46-2^T^	*P*. *granadensis* F-278	*P*. *moraviensis* 1B4^T^	*P*. *koreensis* Ps9-14^T^	*P*.*baetica* a390T	*P*. *vancouverensis* DhA-51^T^	*P*. *jessenii* DSM 17150^T^	*P*. *reinekei* MT1^T^
C10:0 3-OH	5.5	6.6	5.4	3.2	2.6	2.2	3.4	4.8	2.8	3.3
C12:0 2-OH	6.38	5.5	6.8	4.7	4.9	5	5.5	3.8	5.5	4.3
C12:0 3-OH	5.62	6.7	7.5	2.5	4.1	4	3.2	5.7	3.2	4.8
C10:0	ND	0.8	ND	ND	ND	ND	0.1	0.3	0.1	ND
C12:0	1.88	2.9	ND	1.5	2.1	1.6	1.7	3.8	4.7	3.6
C14:0	0.66	0.6	1.2	ND	0.4	0.7	0.5	0.6	0.3	0.7
C16:0	25.07	22.9	33.4	32	29	33	29.4	29.4	29.4	36.5
C17:0 cyclo	0.87	10.3	15.1	6.9	2.4	2	3.2	9.4	0.9	22.3
C18:0	1.35	0.3	1.6	ND	0.5	0.7	0.3	0.2	0.7	0.8
C19:0 ω8c	ND	1.2	ND	ND	0.2	ND	ND	ND	ND	0.7
Summed feature 3	34.78	23.6	16.8	36	36	37	39.5	30.8	38.1	28
Summed feature 8	16.91	13.4	8.9	12	17	13	12.2	8.5	17.2	8.6

Fatty acid composition of ‘*Candidatus* Pseudomonas auctus’ nov. sp. JDE115 and closely related *Pseudomonas* species, analyzed using the Sherlock 6.1 system (Microbial Identification Inc.) and the RTSBA6 library. The values represent the percentage of total fatty acids detected. Summed features refer to groups of two or three fatty acids that cannot be individually resolved using gas chromatography (GC) with the MIDI system. Summed feature 3 includes C16:1ω7c and/or C16:1ω6c, while summed feature 8 comprises C18:1ω7c and/or C18:1ω6c. Data for strain ‘*Candidatus* Pseudomonas auctus’ nov. sp. JDE115 were obtained in this study. Data for other type strains were obtained from references, *P. glycinae* MS586^T^ [73]; *P*. *kribbensis* 46-2^T^ [110]; *P*. *granadensis* F-278,770^T^ [111]; *P*. *moraviensis* 1B4^T^ [111]; *P*. *koreensis* Ps9-14^T^ [112]; *P*.*baetica* a390^T^ [113]; *P*. *vancouverensis* DhA-51^T^ [114]; *P*. *jessenii* DSM 17150^T^ [114]; *P*. *reinekei* MT1^T^ [114].

Abbreviation: ND indicates fatty acids that were not detected.

Compared to closely related species, such as *P. glycinae* MS586T, which shows C16:0 at 22.9%, summed feature 3 at 23.57%, summed feature 8 at 13.37%, and C17:0 cyclo at 10.28%, JDE115 exhibits a higher proportion of summed features 3 and 8 but lower C17:0 cyclo. These differences emphasize the unique chemotaxonomic profile of JDE115 within the genus. Moreover, the characteristic fatty acids C10:0 3-OH, C12:0, and C12:0 3-OH, commonly observed in *Pseudomonas* species, were detected in ‘*Candidatus* P. auctus’ nov. sp*.* JDE115, further validating its classification within the genus.

Strains like *P. kribbensis* 46-2^T^ and *Pseudomonas moraviensis* 1B4^T^ exhibit variations in their fatty acid profiles. For instance, *P. kribbensis* 46-2^T^ has a significantly higher proportion of C16:0 (33.4%) and C17:0 cyclo (15.1%) than JDE115, while *P. moraviensis* 1B4^T^ shows C16:0 at 32% and summed feature 3 at 36%, reflecting its distinct metabolic adaptations (Table 5).

The chemotaxonomic differentiation is further highlighted by unique percentages in summed feature 8 and the presence of minor fatty acids like C19:0 ω8c in JDE115, which were not detected in many other *Pseudomonas* strains. These comparative findings underscore the novelty of ‘*Candidatus* P. auctus’ nov. sp*.* JDE115’s chemotaxonomic profile and its distinct position within the genus.

## Conclusions

Based on a comprehensive polyphasic approach integrating phenotypic, molecular, and chemotaxonomic analyses, ‘*Candidatus* P. auctus’ nov. sp*.* JDE115 is proposed as a novel species within the genus *Pseudomonas*. Its distinct taxonomic position is strongly supported by divergence in genomic similarity indices, including ANI and GGDC, which fall below the species delineation thresholds compared to its closest relatives. Phenotypic characteristics, such as motility, Gram-negative and rod-shaped morphology, facultative aerobiosis, and catalase- and oxidase-positive activity, align with traits typical of the genus while demonstrating unique growth conditions and fluorescence properties. Additionally, the chemotaxonomic profile, including dominant fatty acids such as C16:0, summed feature 3 (C16:1ω7c/C16:1ω6c), and summed feature 8 (C18:1ω7c/C18:1ω6c), further confirms its placement within *Pseudomonas* while distinguishing it from closely related species. These findings underscore the ecological and taxonomic significance of JDE115, providing a strong foundation for exploring its potential in plant growth promotion and biocontrol. The name ‘*Candidatus* P. auctus’ nov. sp*.* is proposed. It remains *candidatus* because the type strain cannot be released to the public because of its potential development as a biopesticide.

### Description of ‘*Candidatus* Pseudomonas auctus’ nov. sp.

*‘Candidatus* P. auctus’ nov. sp. (auc’tus. L. masc. adj. auctus, meaning growth or increase, referring to its ability to promote plant growth) is an aerobic or facultative anaerobic, gram-negative, rod-shaped bacterium with motility conferred by polar flagella. Colonies are fluorescent and appear light-yellow when grown on King’s B agar plates. On LB agar, colonies are smooth, circular, slightly convex, and 5.0–7.5 mm in diameter after 72 hours of incubation at 28°C. Cells are approximately 0.7-.09 X 2.2–3.0 μm in size. Growth occurs at temperatures ranging from 4°C to 40°C, with an optimum temperature of 28–30°C. The bacterium can grow within a pH range of 4.0–10.0, with an optimum pH of 6.0–7.0. ‘*Candidatus* P. auctus’ nov. sp*.* tolerates salinity up to 4% (w/v) NaCl, with optimal growth observed at 1% NaCl.

Biochemical and metabolic characterization using Biolog GENIII Micro Plates (S1 Table) demonstrated the ability of ‘*Candidatus* P. auctus’ nov. sp*.* JDE115 to utilize a variety of substrates. The strain exhibited positive utilization for several carbohydrates, including α-d-glucose, d-mannose, d-fructose, d-galactose, d-fucose, l-fucose, l-rhamnose, and d-mannitol. Amino acid utilization included d-aspartic acid, l-aspartic acid, l-glutamic acid, l-histidine, l-alanine, l-arginine, l-serine, and glycyl-l-proline. The strain also metabolized organic acids such as citric acid, l-malic acid, α-ketoglutaric acid, γ-aminobutyric acid, d-saccharic acid, and acetic acid. ‘*Candidatus* P. auctus’ nov. sp*.* JDE115 demonstrated growth in the presence of chemical inhibitors such as fusidic acid, troleandomycin, rifamycin SV, vancomycin, guanidine HCl, and potassium tellurite, reflecting its adaptive tolerance to diverse chemical conditions. It also exhibited positive results for pH 6 and 5 and tolerated NaCl concentrations up to 4%, but growth was inhibited at 8%. The strain was resistant to aztreonam, nalidixic acid, and lithium chloride but was inhibited by sodium butyrate.

Phylogenetic analysis based on 16S rRNA gene sequences revealed that ‘*Candidatus* P. auctus’ nov. sp*.* JDE115 shares high sequence similarity (>99%) with *P. glycinae* MS586^T^, *P. kribbensis* 46-2^T^, and *P. soyarea* JJL17^T^. However, whole-genome-based ANI (94.59%) and GGDC (57.10%) values with its closest relative, *P. glycinae* MS586^T^, fall below the thresholds for species delineation, confirming its status as a novel species. The strain JDE115 (=SAMN45708326), isolated from soybean root nodules at Kentland Farm, McCoy, Virginia, USA.

## Supporting information

S1 Fig Comparative fluorescence of JDE115 and *Klebsiella* sp. This image highlights the fluorescence capability of ‘*Candidatus* Pseudomonas auctus’ nov. sp. JDE115, evident in three fluorescent plates, compared to the non-fluorescent Klebsiella sp. The test confirms the fluorescence property of JDE115 under UV light.
(TIF)

S1 Movie Live observation of ‘*Candidatus* Pseudomonas auctus’ nov. sp. JDE115 demonstrating active motility. This movie was recorded at 630x using phase contrast, causing the bacteria to appear black; the lighting was gradually changed to bright field to make the bacteria appear white and the clear polysaccharide capsule to become visible.
(MP4)

S2 Fig Morphological characterization of ‘*Candidatus* Pseudomonas auctus’ JDE115 colonies. The diameters of the colonies were measured with a digital caliper after incubation at 28°C on Luria-Bertani agar for 24hr.
(TIF)

S1 Table The physiological, morphological, and phenotypic characteristics of ‘*Candidatus* Pseudomonas auctus’ sp. nov. strain JDE115 in Biology GEN III tests. The tests were repeated three times. This analysis also demonstrated the ability of JDE115 to thrive under aerobic conditions that was evident from the tetrazolium dye reduction assay which functions as an indicator of active respiration. In the BIOLOG system, a positive reaction occurs when the bacterium can metabolize a given substrate, leading to dye reduction and a color change from colorless to purple. The widespread positive metabolic activity across multiple carbon sources confirms the presence of an active electron transport chain (ETC), a hallmark of aerobic respiration. Furthermore, the test results indicated the ability of JDE115 to utilize a broad spectrum of organic acids (e.g., L-malic acid, D-malic acid, citric acid, propionic acid, and acetic acid), which are known to be key carbon sources for aerobic bacteria and facultative anaerobes. This metabolic flexibility indicated a bacterium that efficiently engages in oxidative phosphorylation while still having the capacity to switch to fermentation or alternative electron acceptors when oxygen is limited. The survival of JDE115 under varying NaCl and pH conditions also supports its classification as a facultative aerobe. Its tolerance to 1%, 4%, and 8% NaCl suggests that it can withstand osmotic fluctuations, an important trait for bacteria adapting to different environmental conditions, including those found inside plant tissues.
(DOCX)

S1 File Additional references. (DOCX)
