# Supplementary figures and images for: ‘Candidatus Pseudomonas auctus’ sp. nov. JDE115 isolated from nodules on soybean (Glycines max)
Source: PLoS One. 2025 Sep 11;20(9):e0331920. doi: 10.1371/journal.pone.0331920 (PMC12425225; doi:10.1371/journal.pone.0331920)

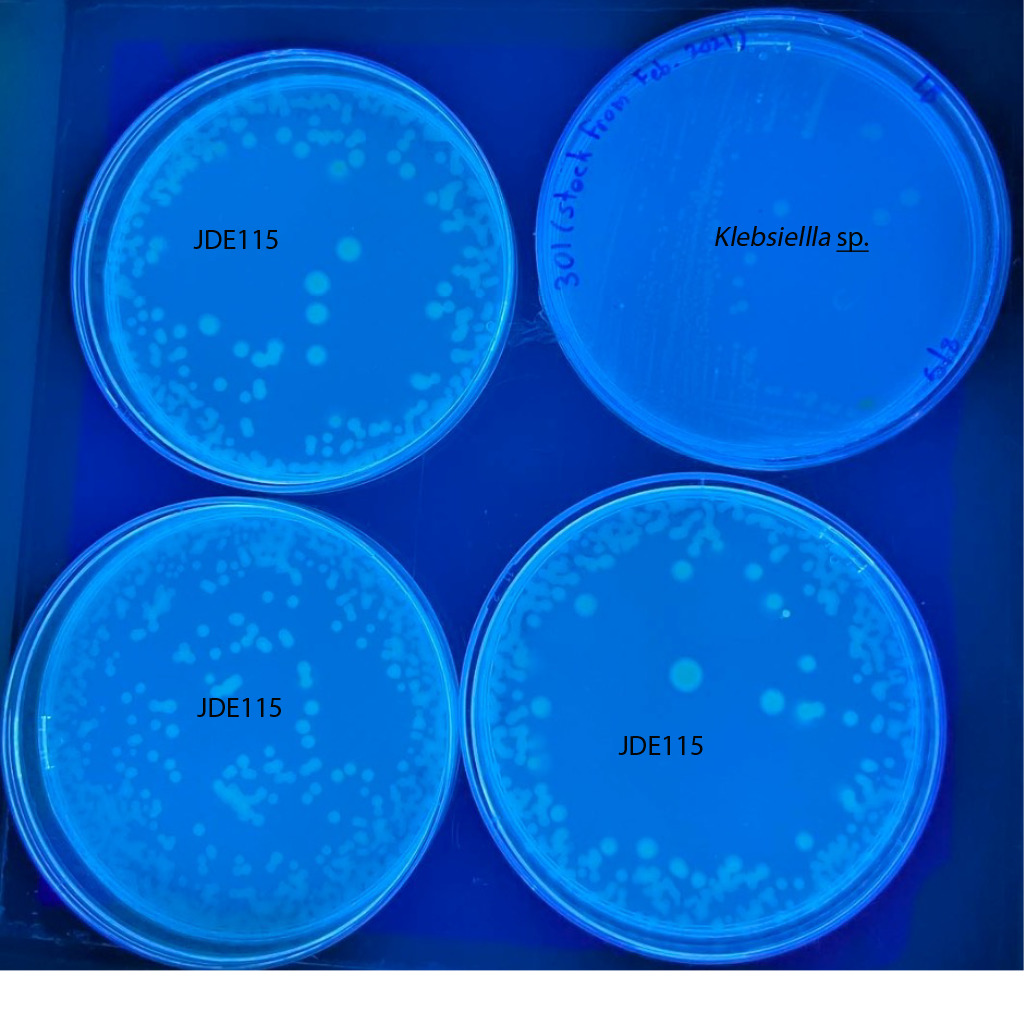

Supplement: S1 Fig — This image highlights the fluorescence capability of ‘Candidatus Pseudomonas auctus’ nov. sp. JDE115, evident in three fluorescent plates, compared to the non-fluorescent Klebsiella sp. The test confirms the fluorescence property of JDE115 under UV light. (TIF) [file pone.0331920.s001.tif]

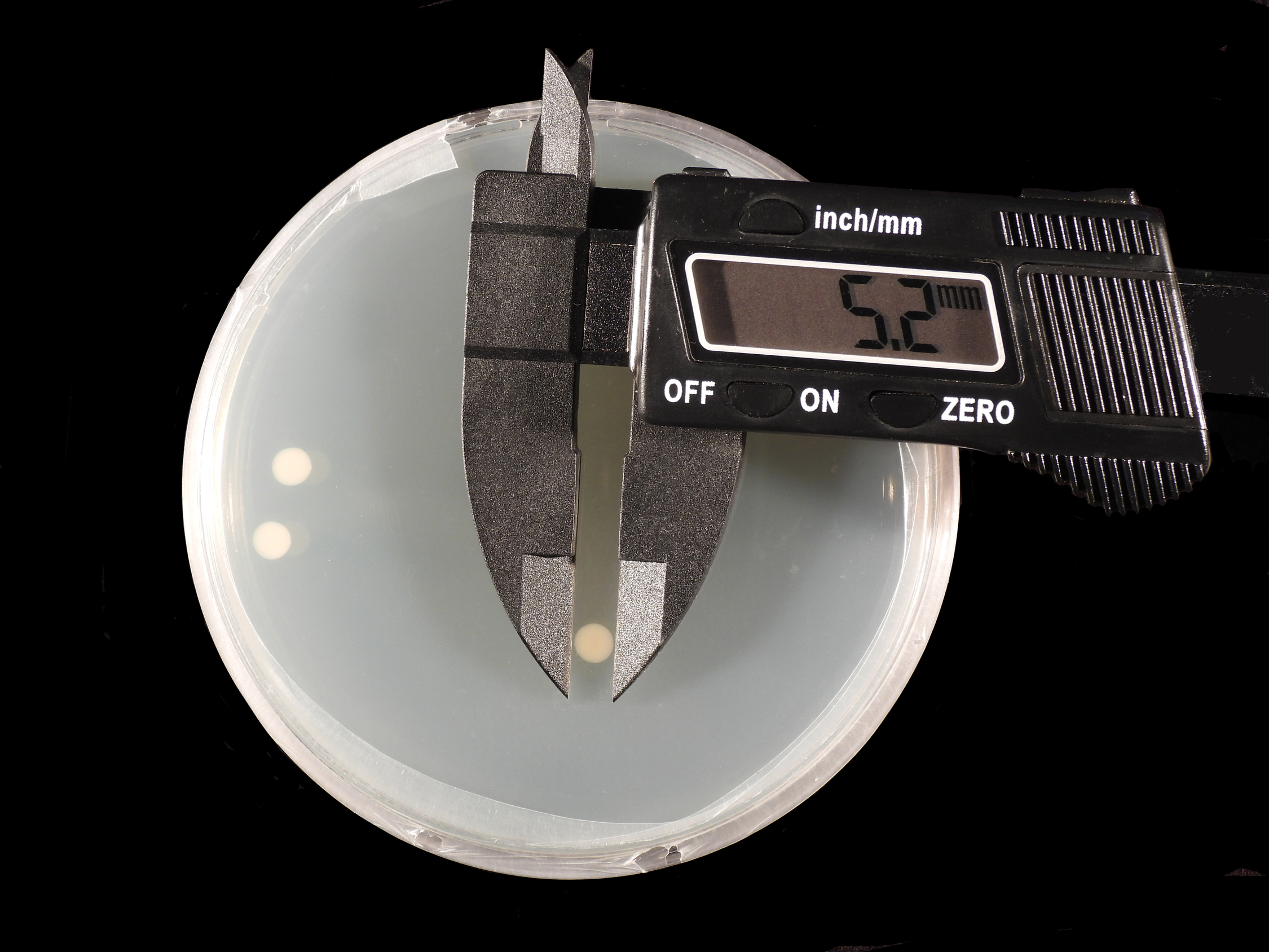

Supplement: S2 Fig — The diameters of the colonies were measured with a digital caliper after incubation at 28°C on Luria-Bertani agar for 24hr. (TIF) [file pone.0331920.s003.tif]
