# Supplementary material for: ‘Candidatus Pseudomonas auctus’ sp. nov. JDE115 isolated from nodules on soybean (Glycines max)
Source: PLoS One. 2025 Sep 11;20(9):e0331920. doi: 10.1371/journal.pone.0331920 (PMC12425225; doi:10.1371/journal.pone.0331920)
Supplement: S1 Table — The tests were repeated three times. This analysis also demonstrated the ability of JDE115 to thrive under aerobic conditions that was evident from the tetrazolium dye reduction assay which functions as an indicator of active respiration. In the BIOLOG system, a positive reaction occurs when the bacterium can metabolize a given substrate, leading to dye reduction and a color change from colorless to purple. The widespread positive metabolic activity across multiple carbon sources confirms the presence of an active electron transport chain (ETC), a hallmark of aerobic respiration. Furthermore, the test results indicated the ability of JDE115 to utilize a broad spectrum of organic acids (e.g., L-malic acid, D-malic acid, citric acid, propionic acid, and acetic acid), which are known to be key carbon sources for aerobic bacteria and facultative anaerobes. This metabolic flexibility indicated a bacterium that efficiently engages in oxidative phosphorylation while still having the capacity to switch to fermentation or alternative electron acceptors when oxygen is limited. The survival of JDE115 under varying NaCl and pH conditions also supports its classification as a facultative aerobe. Its tolerance to 1%, 4%, and 8% NaCl suggests that it can withstand osmotic fluctuations, an important trait for bacteria adapting to different environmental conditions, including those found inside plant tissues. (DOCX) [file pone.0331920.s004.docx]

| **Characteristics** | **reactions** | **Characteristics** | **reactions** | **Characteristics** | **reactions** |
| --- | --- | --- | --- | --- | --- |
| **Negative control** | - | D-Glucose-6-PO4 | - | γ‐Amino‐Butryric Acid | + |
| **Dextrin** | - | D-Fructose-6-PO4 | + | α‐Hydroxy Butyric Acid | - |
| **D-Maltose** | - | D-Aspartic Acid | + | β‐Hydroxy‐D, L‐Butyric  Acid | + |
| **D-Trehalose** | - | D-Serine | + | α‐Keto‐Butyric Acid | - |
| **D-cellobiose** | - | Gelatin | + | Acetoacetic Acid | - |
| **Gentiobiose** | - | Glycyl-L-Proline | + | Propionic Acid | + |
| **Sucrose** | - | L-Alanine | + | Acetic acid | + |
| **D-Turanose** | - | L-Arginine | + | Formic Acid | - |
| **Stachyose** | - | L-Aspartic Acid | + | Positive control | + |
| **D-Raffinose** | - | L-Glutamic Acid | + | pH 6 | + |
| **a-D-Lactose** | - | L-Histidine | + | pH 5 | + |
| **D-Melibiose** | - | L-Pyroglutamic Acid | + | 1%NaCl | + |
| **b-Methyl-D-Glucoside** | - | L-Serine | + | 4%NaCl | + |
| **D-Salicin** | - | Pectin | + | 8%NaCl | - |
| **N-Acetyl-D-Glucosamine** | + | D-Galacturonic acid | + | 1%SodiumLactate | + |
| **N-Acetyl-b-D-Mannosamine** | - | L-Galactonic Acid Lactone | + | Fusidic Acid | + |
| **N-Acetyl-D-Galactosamine** | - | D-Gluconic Acid | + | D-Serine.1 | - |
| **N-Acetyl Neuraminic Acid** | - | D-Glucuronic Acid | + | Troleandomycin | + |
| **a-D-Glucose** | + | Glucuronamide | + | Rifamycin SV | + |
| **D-Mannose** | + | Mucic Acid | + | Minocycline | - |
| **D-Fructose** | + | Quinic Acid | + | Lincomycin | + |
| **D-Galactose** | + | D-Saccharic Acid | + | Guanidine HCl | + |
| **3-Methyl Glucose** | + | p-Hydroxy Phenylacetic Acid | - | Niaproof 4 | + |
| **D-Fucose** | + | Methyl Pyruvate | + | Vancomycin | + |
| **L-Fucose** | + | D-Lactic Acid Methyl Ester | + | Tetrazolium Violet | + |
| **L-Rhamnose** | + | L-Lactic Acid | + | Tetrazolium Blue | + |
| **Inosine** | + | Citric Acid | + | Nalidixic Acid | - |
| **D-Sorbitol** | - | α‐Keto‐Glutaric acid | + | Lithium Chloride | - |
| **D-Mannitol** | + | D-Malic Acid | + | Potassium Tellurite | + |
| **D-Arabitol** | + | L-Malic Acid | + | Aztreonam | - |
| **Myo-Inositol** | + | Bromo-Succinic Acid | + | Sodium Butyrate | - |
| **Glycerol** | + | Tween 40 | + | Sodium Bromate | + |
