# Supplementary material for: ‘Candidatus Pseudomonas auctus’ sp. nov. JDE115 isolated from nodules on soybean (Glycines max)
Source: PLoS One. 2025 Sep 11;20(9):e0331920. doi: 10.1371/journal.pone.0331920 (PMC12425225; doi:10.1371/journal.pone.0331920)
Supplement: S1 File — (DOCX) [file pone.0331920.s005.docx]

1. Migula, W. Über ein neues System der Bakterien. Arb Bakteriol Inst Karlsruhe. 1894;1:235–8.

2. Yang G, Han L, Wen J, Zhou S. *Pseudomonas guangdongensis* sp. nov., isolated from an electroactive biofilm, and emended description of the genus *Pseudomonas* Migula 1894. Int J Syst Evol Microbiol. 2013 Dec 1;63(Pt_12):4599–605.

3. Albert D, Zboralski A, Ciotola M, Cadieux M, Biessy A, Blom J, et al. Identification and genomic characterization of *Pseudomonas* spp. displaying biocontrol activity against *Sclerotinia sclerotiorum* in lettuce. Front Microbiol. 2024 Mar 7;15:1304682.

4. Wang X, Zhou X, Cai Z, Guo L, Chen X, Chen X, et al. A Biocontrol Strain of *Pseudomonas aeruginosa* CQ-40 Promote Growth and Control *Botrytis cinerea* in Tomato. Pathogens. 2020 Dec 31;10(1):22.

5. Duvernoy MC, Mora T, Ardré M, Croquette V, Bensimon D, Quilliet C, et al. Asymmetric adhesion of rod-shaped bacteria controls microcolony morphogenesis. Nat Commun. 2018 Mar 16;9(1):1120.

6. Liu Y, Song Z, Zeng H, Lu M, Zhu W, Wang X, et al. *Pseudomonas eucalypticola* sp. nov., a producer of antifungal agents isolated from *Eucalyptus dunnii* leaves. Sci Rep. 2021 Feb 4;11(1):3006.

7. Krieg NR, Staley JT, Brown DR, Hedlund BP, Paster BJ, Ward NL, et al., editors. Bergey’s Manual^®^ of Systematic Bacteriology: Volume Four The Bacteroidetes, Spirochaetes, Tenericutes (Mollicutes), Acidobacteria, Fibrobacteres, Fusobacteria, Dictyoglomi, Gemmatimonadetes, Lentisphaerae, Verrucomicrobia, Chlamydiae, and Planctomycetes [Internet]. New York, NY: Springer New York; 2010 [cited 2025 Jan 22]. Available from: https://link.springer.com/10.1007/978-0-387-68572-4

8. Shin DH, Choi YS, Cho YH. Unusual Properties of Catalase A (*KatA*) of *Pseudomonas aeruginosa* PA14 Are Associated with Its Biofilm Peroxide Resistance. J Bacteriol. 2008 Apr 15;190(8):2663–70.

9. Su S, Panmanee W, Wilson JJ, Mahtani HK, Li Q, VanderWielen BD, et al. Catalase (KatA) Plays a Role in Protection against Anaerobic Nitric Oxide in *Pseudomonas aeruginosa*. Roop RM, editor. PLoS ONE. 2014 Mar 24;9(3):e91813.

10. Da Cruz Nizer WS, Inkovskiy V, Versey Z, Strempel N, Cassol E, Overhage J. Oxidative Stress Response in *Pseudomonas aeruginosa*. Pathogens. 2021 Sep 14;10(9):1187.

11. Frimmersdorf E, Horatzek S, Pelnikevich A, Wiehlmann L, Schomburg D. How *Pseudomonas aeruginosa* adapts to various environments: a metabolomic approach. Environ Microbiol. 2010 Jun;12(6):1734–47.

12. Kordes A, Preusse M, Willger SD, Braubach P, Jonigk D, Haverich A, et al. Genetically diverse *Pseudomonas aeruginosa* populations display similar transcriptomic profiles in a cystic fibrosis explanted lung. Nat Commun. 2019 Jul 30;10(1):3397.

13. Mei S, Wang M, Salles JF, Hackl T. Diverse rhizosphere-associated *Pseudomonas* genomes from along a Wadden Island salt marsh transition zone. Sci Data. 2024 Oct 17;11(1):1140.

14. Shalev O, Karasov TL, Lundberg DS, Ashkenazy H, Pramoj Na Ayutthaya P, Weigel D. Commensal *Pseudomonas* strains facilitate protective response against pathogens in the host plant. Nat Ecol Evol. 2022 Feb 24;6(4):383–96.

15. Silby MW, Winstanley C, Godfrey SAC, Levy SB, Jackson RW. *Pseudomonas* genomes: diverse and adaptable. FEMS Microbiol Rev. 2011 Jul;35(4):652–80.

16. Thöming JG, Tomasch J, Preusse M, Koska M, Grahl N, Pohl S, et al. Parallel evolutionary paths to produce more than one *Pseudomonas aeruginosa* biofilm phenotype. Npj Biofilms Microbiomes. 2020 Jan 10;6(1):2.

17. Nordstedt NP, Chapin LJ, Taylor CG, Jones ML. Identification of *Pseudomonas* Spp. That Increase Ornamental Crop Quality During Abiotic Stress. Front Plant Sci. 2020 Jan 28;10:1754.

18. Pandey S, Gupta S. Evaluation of *Pseudomonas* sp. for its multifarious plant growth promoting potential and its ability to alleviate biotic and abiotic stress in tomato (*Solanum lycopersicum*) plants. Sci Rep. 2020 Dec 1;10(1):20951.

19. Sah S, Krishnani S, Singh R. *Pseudomonas* mediated nutritional and growth promotional activities for sustainable food security. Curr Res Microb Sci. 2021 Dec;2:100084.

20. Zheng Y, Cao X, Zhou Y, Ma S, Wang Y, Li Z, et al. Purines enrich root-associated *Pseudomonas* and improve wild soybean growth under salt stress. Nat Commun. 2024 Apr 25;15(1):3520.

21. Qin S, Xiao W, Zhou C, Pu Q, Deng X, Lan L, et al. *Pseudomonas aeruginosa*: pathogenesis, virulence factors, antibiotic resistance, interaction with host, technology advances and emerging therapeutics. Signal Transduct Target Ther. 2022 Jun 25;7(1):199.

22. Bakki M, Banane B, Marhane O, Esmaeel Q, Hatimi A, Barka EA, et al. Phosphate solubilizing *Pseudomonas* and *Bacillus* combined with rock phosphates promoting tomato growth and reducing bacterial canker disease. Front Microbiol. 2024 May 3;15:1289466.

23. Blanco-Vargas A, Rodríguez-Gacha LM, Sánchez-Castro N, Garzón-Jaramillo R, Pedroza-Camacho LD, Poutou-Piñales RA, et al. Phosphate-solubilizing *Pseudomonas* sp., and Serratia sp., co-culture for *Allium cepa* L. growth promotion. Heliyon. 2020 Oct;6(10):e05218.

24. Chen J, Zhao G, Wei Y, Dong Y, Hou L, Jiao R. Isolation and screening of multifunctional phosphate solubilizing bacteria and its growth-promoting effect on Chinese fir seedlings. Sci Rep. 2021 Apr 27;11(1):9081.

25. Faller L, Leite MFA, Kuramae EE. Enhancing phosphate-solubilising microbial communities through artificial selection. Nat Commun. 2024 Feb 23;15(1):1649.

26. Ou K, He X, Cai K, Zhao W, Jiang X, Ai W, et al. Phosphate-Solubilizing *Pseudomonas* sp. Strain WS32 Rhizosphere Colonization-Induced Expression Changes in Wheat Roots. Front Microbiol. 2022 Jun 30;13:927889.

27. Paul D, Sinha SN. Isolation and characterization of phosphate solubilizing bacterium *Pseudomonas aeruginosa* KUPSB12 with antibacterial potential from river Ganga, India. Ann Agrar Sci. 2017 Mar;15(1):130–6.

28. Grosse C, Brandt N, Van Antwerpen P, Wintjens R, Matthijs S. Two new siderophores produced by *Pseudomonas* sp. NCIMB 10586: The anti-oomycete non-ribosomal peptide synthetase-dependent mupirochelin and the NRPS-independent triabactin. Front Microbiol. 2023 Mar 24;14:1143861.

29. Jin Z, Li J, Ni L, Zhang R, Xia A, Jin F. Conditional privatization of a public siderophore enables *Pseudomonas aeruginosa* to resist cheater invasion. Nat Commun. 2018 Apr 11;9(1):1383.

30. Luján AM, Gómez P, Buckling A. Siderophore cooperation of the bacterium *Pseudomonas fluorescens* in soil. Biol Lett. 2015 Feb;11(2):20140934.

31. Song Y, Wu X, Li Z, Ma QQ, Bao R. Molecular mechanism of siderophore regulation by the *Pseudomonas aeruginosa* BfmRS two-component system in response to osmotic stress. Commun Biol. 2024 Mar 9;7(1):295.

32. Syed A, Elgorban AM, Bahkali AH, Eswaramoorthy R, Iqbal RK, Danish S. Metal-tolerant and siderophore producing *Pseudomonas* fluorescence and *Trichoderma* spp. improved the growth, biochemical features and yield attributes of chickpea by lowering Cd uptake. Sci Rep. 2023 Mar 18;13(1):4471.

33. Chi SI, Akuma M, Xu R, Plante V, Hadinezhad M, Tambong JT. Phenazines are involved in the antagonism of a novel subspecies of *Pseudomonas chlororaphis* strain S1Bt23 against *Pythium ultimum*. Sci Rep. 2024 Sep 3;14(1):20517.

34. Garbeva P, Silby MW, Raaijmakers JM, Levy SB, De Boer W. Transcriptional and antagonistic responses of *Pseudomonas fluorescens* Pf0-1 to phylogenetically different bacterial competitors. ISME J. 2011 Jun 1;5(6):973–85.

35. Mehmood N, Saeed M, Zafarullah S, Hyder S, Rizvi ZF, Gondal AS, et al. Multifaceted Impacts of Plant-Beneficial *Pseudomonas* spp. in Managing Various Plant Diseases and Crop Yield Improvement. ACS Omega. 2023 Jun 27;8(25):22296–315.

36. Neve RL, Giedraitis E, Akbari MS, Cohen S, Phelan VV. Secondary metabolite profiling of *Pseudomonas aeruginosa* isolates reveals rare genomic traits. Porto C, editor. mSystems. 2024 May 16;9(5):e00339-24.

37. Gomila M, PeÃ±a A, Mulet M, Lalucat J, GarcÃ­a-ValdÃ©s E. Phylogenomics and systematics in *Pseudomonas*. Front Microbiol [Internet]. 2015 Mar 18 [cited 2025 Jan 22];6. Available from: http://www.frontiersin.org/Evolutionary_and_Genomic_Microbiology/10.3389/fmicb.2015.00214/abstract

38. Gomila M, Busquets A, Mulet M, García-Valdés E, Lalucat J. Clarification of Taxonomic Status within the *Pseudomonas syringae* Species Group Based on a Phylogenomic Analysis. Front Microbiol. 2017 Dec 7;8:2422.

39. Hu S, Li X, Yin X, Li R, Zhang R, Zang J, et al. Species-specific identification of *Pseudomonas* based on 16S–23S rRNA gene internal transcribed spacer (ITS) and its combined application with next-generation sequencing. BMC Microbiol. 2022 Dec;22(1):188.

40. Scales BS, Dickson RP, LiPuma JJ, Huffnagle GB. Microbiology, Genomics, and Clinical Significance of the *Pseudomonas fluorescens* Species Complex, an Unappreciated Colonizer of Humans. Clin Microbiol Rev. 2014 Oct;27(4):927–48.

41. Ferrer Obiol J, Herranz JM, Paris JR, Whiting JR, Rozas J, Riutort M, et al. Species delimitation using genomic data to resolve taxonomic uncertainties in a speciation continuum of pelagic seabirds. Mol Phylogenet Evol. 2023 Feb;179:107671.

42. Lalucat J, Mulet M, Gomila M, García-Valdés E. Genomics in Bacterial Taxonomy: Impact on the Genus *Pseudomonas*. Genes. 2020 Jan 29;11(2):139.

43. Puigbò P, Wolf YI, Koonin EV. Genome-Wide Comparative Analysis of Phylogenetic Trees: The Prokaryotic Forest of Life. In: Anisimova M, editor. Evolutionary Genomics [Internet]. Totowa, NJ: Humana Press; 2012 [cited 2025 Jan 22]. p. 53–79. (Methods in Molecular Biology; vol. 856). Available from: https://link.springer.com/10.1007/978-1-61779-585-5_3

44. Jain C, Rodriguez-R LM, Phillippy AM, Konstantinidis KT, Aluru S. High throughput ANI analysis of 90K prokaryotic genomes reveals clear species boundaries. Nat Commun. 2018 Nov 30;9(1):5114.

45. Richter M, Rosselló-Móra R. Shifting the genomic gold standard for the prokaryotic species definition. Proc Natl Acad Sci. 2009 Nov 10;106(45):19126–31.

46. Rodriguez-R LM, Conrad RE, Viver T, Feistel DJ, Lindner BG, Venter SN, et al. An ANI gap within bacterial species that advances the definitions of intra-species units. Jouline IB, editor. mBio. 2024 Jan 16;15(1):e02696-23.

47. Thompson CC, Chimetto L, Edwards RA, Swings J, Stackebrandt E, Thompson FL. Microbial genomic taxonomy. BMC Genomics. 2013; 14: 913. doi:10.1186/1471-2164-14-913.

48. Auch AF, von Jan M, Klenk HP, Göker M. Digital DNA-DNA hybridization for microbial species delineation by means of genome-to-genome sequence comparison. Standards in Genomic Sciences. 2010; 2(1):117–34. doi:10.4056/sigs.531120

49. Meier-Kolthoff JP, Auch AF, Klenk HP, Göker M. Genome sequence-based species delimitation with confidence intervals and improved distance functions. BMC Bioinformatics. 2013 Dec;14(1):60.

50. Auch AF, Klenk HP, Göker M. Standard operating procedure for calculating genome-to-genome distances based on high-scoring segment pairs. Stand Genomic Sci. 2010 Jan 28;2(1):142–8.

51. Church DL, Cerutti L, Gürtler A, Griener T, Zelazny A, Emler S. Performance and Application of 16S rRNA Gene Cycle Sequencing for Routine Identification of Bacteria in the Clinical Microbiology Laboratory. Clin Microbiol Rev. 2020 Sep 16;33(4):e00053-19.

52. Clarridge JE. Impact of 16S rRNA Gene Sequence Analysis for Identification of Bacteria on Clinical Microbiology and Infectious Diseases. Clin Microbiol Rev. 2004 Oct;17(4):840–62.

53. Hassler HB, Probert B, Moore C, Lawson E, Jackson RW, Russell BT, et al. Phylogenies of the 16S rRNA gene and its hypervariable regions lack concordance with core genome phylogenies. Microbiome. 2022 Dec;10(1):104.

54. Gabriel MW, Matsui GY, Friedman R, Lovell CR. Optimization of Multilocus Sequence Analysis for Identification of Species in the Genus *Vibrio*. Elkins CA, editor. Appl Environ Microbiol. 2014 Sep;80(17):5359–65.

55. Korczak B, Christensen H, Emler S, Frey J, Kuhnert P. Phylogeny of the family Pasteurellaceae based on *rpoB* sequences. Int J Syst Evol Microbiol. 2004 Jul 1;54(4):1393–9.

56. López-Hermoso C, De La Haba RR, Sánchez-Porro C, Papke RT, Ventosa A. Assessment of MultiLocus Sequence Analysis as a Valuable Tool for the Classification of the Genus *Salinivibrio*. Front Microbiol. 2017 Jun 22;8:1107.

57. Mulet M, Bennasar A, Lalucat J, García-Valdés E. An *rpoD*-based PCR procedure for the identification of *Pseudomonas* species and for their detection in environmental samples. Mol Cell Probes. 2009 Jun;23(3–4):140–7.

58. Yáñez MA, Catalán V, Apráiz D, Figueras MJ, Martínez-Murcia AJ. Phylogenetic analysis of members of the genus *Aeromonas* based on *gyrB* gene sequences. Int J Syst Evol Microbiol. 2003 May 1;53(3):875–83.

59. Moule AL, Wilkinson SG. Polar Lipids, Fatty Acids, and Isoprenoid Quinones of *Alteromonas putrefaciens* (*Shewanella putrefaciens*). Syst Appl Microbiol. 1987 Aug;9(3):192–8.

60. Yamano R, Yu J, Jiang C, Harjuno Condro Haditomo A, Mino S, Sakai Y, et al. Taxonomic revision of the genus *Amphritea* supported by genomic and in silico chemotaxonomic analyses, and the proposal of *Aliamphritea* gen. nov. Yurchenko V, editor. PLOS ONE. 2022 Aug 10;17(8):e0271174.

61. Zou Y, Lin X, Xue W, Tuo L, Chen MS, Chen XH, et al. Characterization and description of *Faecalibacterium butyricigenerans* sp. nov. and *F. longum* sp. nov., isolated from human faeces. Sci Rep. 2021 May 31;11(1):11340.

62. Lacey RF, Sullivan-Hill BA, Deslippe JR, Keyzers RA, Gerth ML. The Fatty Acid Methyl Ester (FAME) profile of *Phytophthora agathidicida* and its potential use as diagnostic tool. FEMS Microbiol Lett. 2021 Sep 8;368(17):fnab113.

63. Heyrman J, Mergaert J, Denys R, Swings J. The use of fatty acid methyl ester analysis (FAME) for the identification of heterotrophic bacteria present on three mural paintings showing severe damage by microorganisms. FEMS Microbiol Lett. 1999 Dec;181(1):55–62.

64. Inglis TJJ, Aravena-Roman M, Ching S, Croft K, Wuthiekanun V, Mee BJ. Cellular Fatty Acid Profile Distinguishes *Burkholderia pseudomallei* from Avirulent *Burkholderia thailandensis*. J Clin Microbiol. 2003 Oct;41(10):4812–4.

65. Rees CA, Beccaria M, Franchina FA, Hill JE, Purcaro G. Fatty Acid Methyl Ester (FAME) Profiling Identifies Carbapenemase-Producing *Klebsiella pneumoniae* Belonging to Clonal Complex 258. Separations. 2019 Jun 17;6(2):32.

66. Vauterin L, Yang P, Swings J. Utilization of Fatty Acid Methyl Esters for the Differentiation of New *Xanthomonas* Species. Int J Syst Bacteriol. 1996 Jan 1;46(1):298–304.

67. Kunitsky C, Osterhout G, Sasser M. Identification of microorganisms using fatty acid methyl ester (FAME) analysis and the MIDI Sherlock^®^ microbial identification system. Newark (DE): MIDI, Inc.; 2006.

68. Kim M, Song J, Shin SY, Kogure K, Kang I, Cho JC. Cultivation of deep-sea bacteria from the Northwest Pacific Ocean and characterization of *Limnobacter profundi* sp. nov., a phenol-degrading bacterium. Front Mar Sci. 2024 Nov 4;11:1449548.

69. Tareen S, Risdian C, Müsken M, Wink J. *Qipengyuania pacifica* sp. nov., a Novel Carotenoid-Producing Marine Bacterium of the Family Erythrobacteraceae, Isolated from Sponge (Demospongiae), and Antimicrobial Potential of Its Crude Extract. Diversity. 2022 Apr 14;14(4):295.

70. Yin LZ, Li JL, Fang BZ, Liu ZT, Wang P, Dong L, et al. *Roseomonas ponticola* sp. nov., a novel bacterium isolated from Pearl River estuary. Int J Syst Evol Microbiol [Internet]. 2021 Oct 20 [cited 2025 Jan 22];71(10).

71. Yin LZ, Li JL, Fang BZ, Liu ZT, Wang P, Dong L, et al. *Roseomonas ponticola* sp. nov., a novel bacterium isolated from Pearl River estuary. Int J Syst Evol Microbiol [Internet]. 2021 Oct 20 [cited 2025 Jan 22];71(10).

72. Matz C, Deines P, JÃ¼rgens K. Phenotypic variation in *Pseudomonas* sp. CM10 determines microcolony formation and survival under protozoan grazing. FEMS Microbiol Ecol. 2002 Jan;39(1):57–65.

73. Orellana-Saez M, Pacheco N, Costa JI, Mendez KN, Miossec MJ, Meneses C, et al. In-Depth Genomic and Phenotypic Characterization of the Antarctic Psychrotolerant Strain *Pseudomonas* sp. MPC6 Reveals Unique Metabolic Features, Plasticity, and Biotechnological Potential. Front Microbiol. 2019 May 24;10:1154.

74. Jia J, Wang X, Deng P, Ma L, Baird SM, Li X, et al. *Pseudomonas glycinae* sp. nov. isolated from the soybean rhizosphere. MicrobiologyOpen. 2020 Sep;9(9):e1101.

75. Bouteiller M, Dupont C, Bourigault Y, Latour X, Barbey C, Konto-Ghiorghi Y, et al. *Pseudomonas Flagella*: Generalities and Specificities. Int J Mol Sci. 2021 Mar 24;22(7):3337.

76. Kearns DB. A field guide to bacterial swarming motility. Nat Rev Microbiol. 2010 Sep;8(9):634–44.

77. Kühn MJ, Schmidt FK, Farthing NE, Rossmann FM, Helm B, Wilson LG, et al. Spatial arrangement of several flagellins within bacterial flagella improves motility in different environments. Nat Commun. 2018 Dec 18;9(1):5369.

78. Bartholomew JW, Mittwer T. The Gram Stain. Bacteriol Rev. 1952 Mar;16(1):1–29.

79. Mrazova K, Bacovsky J, Sedrlova Z, Slaninova E, Obruca S, Fritz I, et al. Urany-Less Low Voltage Transmission Electron Microscopy: A Powerful Tool for Ultrastructural Studying of Cyanobacterial Cells. Microorganisms. 2023 Mar 29;11(4):888.

80. Crespi R, Barone A, Covani U, Ciaglia RN, Romanos GE. Effects of CO_2_ Laser Treatment on Fibroblast Attachment to Root Surfaces. A Scanning Electron Microscopy Analysis. J Periodontol. 2002 Nov;73(11):1308–12.

81. Siqueira JF, Lopes HP. Bacteria on the apical root surfaces of untreated teeth with periradicular lesions: a scanning electron microscopy study. Int Endod J. 2001 Apr;34(3):216–20.

82. Shotton DM. Review: Video-enhanced light microscopy and its applications in cell biology. J Cell Sci. 1988 Feb 1;89(2):129–50.

83. Lamichhane JR, Varvaro L. A new medium for the detection of fluorescent pigment production by pseudomonads. Plant Pathol. 2013 Jun;62(3):624–32.

84. Mira P, Yeh P, Hall BG. Estimating microbial population data from optical density. Omri A, editor. PLOS ONE. 2022 Oct 13;17(10):e0276040.

85. Sezonov G, Joseleau-Petit D, D’Ari R. *Escherichia coli* Physiology in Luria-Bertani Broth. J Bacteriol. 2007 Dec;189(23):8746–9.

86. Zhang K, Ding W, Han C, Long L, Yin H, Yin J. Investigation on taxonomy, secondary metabolites and antibacterial activity of *Streptomyces sediminicola* sp. nov., a novel marine sediment-derived Actinobacteria. Microb Cell Factories. 2024 Oct 19;23(1):285.

**Additional references**

**P1. Ali MS, Mony FTZ, Eisenback JD.** Surface Sterilization of Soybean Nodules for Microbiological Studies. protocols.io, 2025a. <https://www.protocols.io/private/3F5FACE8049811F0B4580A58A9FEAC02>

**P2. Ali MS, Mony FTZ, Eisenback JD.** Selective King’s B Medium for Gram Negative Bacteria. protocols.io, 2025b. <https://www.protocols.io/private/9FFDB692048E11F0A6C00A58A9FEAC02>

**P3. Ali MS, Mony FTZ, Eisenback JD.** LB lite Growth Media Protocol. protocols.io, 2025c. https://www.protocols.io/private/E2F913ED044011F0A7330A58A9FEAC02

**P4. Ali MS, Mony FTZ, Eisenback JD.** Glycerol Stock Solution Preparation for Long-Term Bacterial Storage. protocols.io, 2025d. https://www.protocols.io/private/5C456094049E11F0A6C00A58A9FEAC02

**P5. Ali MS, Mony FTZ, Eisenback JD.**Transmission Electron Microscopy (TEM) Protocol for Pseudomonas sp. protocols.io, 2025f. https://www.protocols.io/private/A9FB11B9055B11F0B0AB0A58A9FEAC02

**P6. Ali MS, Mony FTZ, Eisenback JD.** Identification of *Pseudomonas* sp. using fatty acid methyl ester (FAME) analysis. protocols.io, 2025g. https://www.protocols.io/private/714A65A7056511F0B0AB0A58A9FEAC02

**P7. Ali MS, Mony FTZ, Eisenback JD.** Identification of Bacteria using BIOLOG GEN III Assay. protocols.io, 2025h. https://www.protocols.io/private/B4B9CBFA059511F082780A58A9FEAC02

**P8. Ali MS, Mony FTZ, Eisenback JD.** Genomic DNA Extraction Protocol using DNeasy® UltraClean® Microbial Kit. protocols.io, 2025i. https://www.protocols.io/private/4D8344B004F311F0B0AB0A58A9FEAC02

**P9. Ali MS, Mony FTZ, Eisenback JD.** Agarose Gel Electrophoresis for DNA Analysis. protocols.io, 2025j. https://www.protocols.io/private/801EE22204E211F0A6C00A58A9FEAC02

**P10. Ali MS, Mony FTZ, Eisenback JD.** PCR Run Protocol for Bacterial 16S rRNA Gene Amplification. protocols.io, 2025k. https://www.protocols.io/private/C8D78DC0055411F0B0AB0A58A9FEAC02
