# Supplementary material for: ‘Candidatus Pseudomonas auctus’ sp. nov. JDE115 isolated from nodules on soybean (Glycines max)
Source: PLoS One. 2025 Sep 11;20(9):e0331920. doi: 10.1371/journal.pone.0331920 (PMC12425225; doi:10.1371/journal.pone.0331920)
Supplement: Attachment — Submitted filename: PONE-D-25-28050.pdf [file pone.0331920.s006.pdf]

PLOS ONE

‘Candidatus Pseudomonas auctus’ sp. nov. JDE115 isolated from nodules on soybean (Glycines max)

--Manuscript Draft--

**Manuscript Number:**	PONE-D-25-28050
**Article Type:**	Research Article
**Full Title:**	‘Candidatus Pseudomonas auctus’ sp. nov. JDE115 isolated from nodules on soybean (Glycines max)
**Short Title:**	'Candidatus Pseudomonas auctus sp. nov. description
**Corresponding Author:**	Jonathan D. Eisenback, Ph. D.<br>Virginia Tech: Virginia Polytechnic Institute and State University<br>Blacksburg, UNITED STATES OF AMERICA
**Keywords:**	Candidatus Pseudomonas auctus; Pseudomonas; Soybean root nodules; Novel species; 16S rRNA; Whole-Genome Sequencing; Average Nucleotide Identity (ANI); Genome-to-Genome Distance Calculator (GGDC); Plant growth-promoting bacteria; Chemotaxonomy; Biocontrol agent; Fluorescent pseudomonads; Fatty acid methyl ester (FAME) analysis; Nitrogen fixation; Phylogenomic analysis; Soybean; agriculture; Biofertilizer
**Abstract:**	A Gram-negative, facultative anaerobic, rod-shaped, motile with peritrichous flagella, fluorescent bacterium, designated ‘Candidatus Pseudomonas auctus’ sp. nov. JDE115, was isolated from soybean root nodules in Virginia and characterized using a comprehensive integrative methodology. Growth of JDE115 occurred with 0–5.0% (w/v) NaCl (optimum 1%), at pH 6.0–10.0 (optimum pH 7.0), and at 10–40°C (optimum 28°C) in LB broth. Phylogenetic analyses based on the 16S rRNA gene placed the isolate as a member of a novel species within the genus Pseudomonas. Phylogenetic analyses based on whole-genome sequences, 16S rRNA, showed JDE115 having the highest similarity to Pseudomonas glycinae MS586. Average Nucleotide Identity (ANI) analysis also revealed the highest similarity of JDE115 to Pseudomonas glycinae MS586 (94.59%), which is below the 95% threshold for species delineation. Genome-to-genome distance analysis (GGDC, Formula 2) showed a maximum value of 57.10% with the same strain, far below the 70% cutoff. The primary isoprenoid quinone detected in JDE115 was ubiquinone-9 (Q-9) and the DNA G+C content was 60.68 mol%. The whole-cell fatty acid profile was dominated by C16:0, C17:0 cyclo, and the summed features 3 (C16:1ω7c and/or C16:1ω6c) and 8 (C18:1ω7c and/or C18:1ω6c). Additional fatty acids detected included 12:0, 14:0, and 18:0. Based on these phenotypic, chemotaxonomic, and phylogenetic data, strain JDE115 is proposed to represent a new species in the genus Pseudomonas, for which the name ‘Candidatus Pseudomonas auctus’ sp. nov. is proposed.
**Order of Authors:**	Jonathan D. Eisenback, Ph. D.
	Md Sahadat Ali, PhD
	Fatima Tuz Zohora Mony, MS
	Michael Evans, PhD
	Steven Rideout, PhD
	David Haak, PhD
	Paulo Vieira, PhD
**Additional Information:**	
**Question**	**Response**
**Financial Disclosure**<br><br>Enter a financial disclosure statement that describes the sources of funding for the	Yes

work included in this submission. Review the submission guidelines for detailed requirements. View published research articles from *PLOS ONE* for specific examples.<br><br>This statement is required for submission and **will appear in the published article** if the submission is accepted. Please make sure it is accurate.<br><br>**Funded studies**<br>Enter a statement with the following details:<br>• Initials of the authors who received each award<br>• Grant numbers awarded to each author<br>• The full name of each funder<br>• URL of each funder website<br>• Did the sponsors or funders play any role in the study design, data collection and analysis, decision to publish, or preparation of the manuscript?<br><br>Did you receive funding for this work?	
Please add funding details.<br>as follow-up to "**Financial Disclosure**<br><br>Enter a financial disclosure statement that describes the sources of funding for the work included in this submission. Review the submission guidelines for detailed requirements. View published research articles from *PLOS ONE* for specific examples.<br><br>This statement is required for submission and **will appear in the published article** if the submission is accepted. Please make sure it is accurate.	Virginia Tech Intellectual Property LAUNCH Proof-of-Concept Grant<br>The School of Plant and Environmental Sciences, Virginia Tech research associate and tuition

Powered by Editorial Manager® and ProduXion Manager® from Aries Systems Corporation

**Funded studies**<br>Enter a statement with the following details:<br>• Initials of the authors who received each award<br>• Grant numbers awarded to each author<br>• The full name of each funder<br>• URL of each funder website<br>• Did the sponsors or funders play any role in the study design, data collection and analysis, decision to publish, or preparation of the manuscript?<br><br>Did you receive funding for this work?"	
Please select the country of your main research funder (please select carefully as in some cases this is used in fee calculation).<br>as follow-up to "**Financial Disclosure**<br><br>Enter a financial disclosure statement that describes the sources of funding for the work included in this submission. Review the submission guidelines for detailed requirements. View published research articles from *PLOS ONE* for specific examples.<br><br>This statement is required for submission and **will appear in the published article** if the submission is accepted. Please make sure it is accurate.<br><br>**Funded studies**<br>Enter a statement with the following details:<br>• Initials of the authors who received each award<br>• Grant numbers awarded to each author<br>• The full name of each funder<br>• URL of each funder website<br>• Did the sponsors or funders play any role in the study design, data collection and analysis, decision to publish, or preparation of the manuscript?<br><br>Did you receive funding for this work?"	UNITED STATES - US
**Competing Interests**	The authors have declared that no competing interests exist.

Use the instructions below to enter a competing interest statement for this submission. On behalf of all authors, disclose any competing interests that could be perceived to bias this work—acknowledging all financial support and any other relevant financial or non-financial competing interests.<br><br>This statement is required for submission and **will appear in the published article** if the submission is accepted. Please make sure it is accurate and that any funding sources listed in your Funding Information later in the submission form are also declared in your Financial Disclosure statement.<br><br>View published research articles from *PLOS ONE* for specific examples.<br><br>**NO authors have competing interests**<br><br>Enter: *The authors have declared that no competing interests exist.*<br><br>**Authors with competing interests**<br><br>Enter competing interest details beginning with this statement:<br><br>*I have read the journal's policy and the authors of this manuscript have the following competing interests: [insert competing interests here]*<br><br>* typeset	
**Ethics Statement**<br><br>Enter an ethics statement for this submission. This statement is required if the study involved:<br><br>• Human participants<br>• Human specimens or tissue<br>• Vertebrate animals or cephalopods<br>• Vertebrate embryos or tissues	N/A

• Field research<br><br>Write "N/A" if the submission does not require an ethics statement.<br><br>General guidance is provided below. Consult the submission guidelines for detailed instructions. **Make sure that all information entered here is included in the Methods section of the manuscript.**	

**Format for specific study types**<br><br>**Human Subject Research (involving human participants and/or tissue)**<br>• Give the name of the institutional review board or ethics committee that approved the study<br>• Include the approval number and/or a statement indicating approval of this research<br>• Indicate the form of consent obtained (written/oral) or the reason that consent was not obtained (e.g. the data were analyzed anonymously)<br><br>**Animal Research (involving vertebrate animals, embryos or tissues)**<br>• Provide the name of the Institutional Animal Care and Use Committee (IACUC) or other relevant ethics board that reviewed the study protocol, and indicate whether they approved this research or granted a formal waiver of ethical approval<br>• Include an approval number if one was obtained<br>• If the study involved *non-human primates*, add *additional details* about animal welfare and steps taken to ameliorate suffering<br>• If anesthesia, euthanasia, or any kind of animal sacrifice is part of the study, include briefly which substances and/or methods were applied<br><br>**Field Research**<br><br>Include the following details if this study involves the collection of plant, animal, or other materials from a natural setting:<br>• Field permit number<br>• Name of the institution or relevant body that granted permission	
**Data Availability**<br><br>Authors are required to make all data underlying the findings described fully available, without restriction, and from the time of publication. PLOS allows rare exceptions to address legal and ethical concerns. See the PLOS Data Policy and FAQ for detailed information.	Yes - all data are fully available without restriction

A Data Availability Statement describing where the data can be found is required at submission. Your answers to this question constitute the Data Availability Statement and **will be published in the article**, if accepted.<br><br>**Important:** Stating 'data available on request from the author' is not sufficient. If your data are only available upon request, select 'No' for the first question and explain your exceptional situation in the text box.<br><br>Do the authors confirm that all data underlying the findings described in their manuscript are fully available without restriction?	
**Describe where the data may be found in full sentences. If you are copying our sample text, replace any instances of XXX with the appropriate details.**<br><br>• If the data are **held or will be held in a public repository**, include URLs, accession numbers or DOIs. If this information will only be available after acceptance, indicate this by ticking the box below. For example: *All XXX files are available from the XXX database (accession number(s) XXX, XXX.*).<br>• If the data are all contained **within the manuscript and/or Supporting Information files**, enter the following: *All relevant data are within the manuscript and its Supporting Information files.*<br>• If neither of these applies but you are able to provide **details of access elsewhere**, with or without limitations, please do so. For example:<br><br>*Data cannot be shared publicly because of [XXX]. Data are available from the XXX Institutional Data Access / Ethics Committee (contact via XXX) for researchers who meet the criteria for access to confidential data.*<br><br>*The data underlying the results presented in the study are available from (include the name of the third party*	All relevant data are within the manuscript and its Supporting Information files.

and contact information or URL).<br>• This text is appropriate if the data are owned by a third party and authors do not have permission to share the data.<br><br>* typeset	
Additional data availability information:	

Powered by Editorial Manager® and ProduXion Manager® from Aries Systems Corporation

Manuscript
Click here to access/download;Manuscript;Candidatus P. auctus_05_29_25_Ali_Final.docx

‘*Candidatus* Pseudomonas auctus’ sp. nov. JDE115 isolated from nodules on soybean (*Glycines max*)

Md Sahadat Ali[1¶], Fatima Tuz Zohora Mony[1&], Michael Evans[1&], Steven Rideout[1&], David Haak[1&], Paulo Vieira[2&], Jonathan D. Eisenback[1¶*]

[1]School of Plant and Environmental Sciences, Virginia Tech, Blacksburg, Virginia, United States of America

[2] Mycology and Nematology Genetic Diversity and Biology Laboratory, United States Department of Agriculture, Agricultural Research Service, Beltsville, Maryland, United States of America

*****Corresponding author

E-mail: jon@vt.edu (JE)

[¶]SA and JE are Joint Senior Authors

[&]These authors contributed equally to this work

Abstract

A Gram-negative, facultative anaerobic, rod-shaped, motile with peritrichous flagella, fluorescent bacterium, designated ‘*Candidatus* Pseudomonas auctus’ sp. nov. JDE115, was isolated from soybean root nodules in Virginia and characterized using a comprehensive integrative methodology. Growth of JDE115 occurred with 0–5.0% (w/v) NaCl (optimum 1%), at pH 6.0–10.0 (optimum pH 7.0), and at 10–40°C (optimum 28°C) in LB broth. Phylogenetic

analyses based on the 16S rRNA gene placed the isolate as a member of a novel species within the genus *Pseudomonas.* Phylogenetic analyses based on whole-genome sequences, 16S rRNA, showed JDE115 having the highest similarity to *Pseudomonas glycinae* MS586. Average Nucleotide Identity (ANI) analysis also revealed the highest similarity of JDE115 to *Pseudomonas glycinae* MS586 (94.59%), which is below the 95% threshold for species delineation. Genome-to-genome distance analysis (GGDC, Formula 2) showed a maximum value of 57.10% with the same strain, far below the 70% cutoff. The primary isoprenoid quinone detected in JDE115 was ubiquinone-9 (Q-9) and the DNA G+C content was 60.68 mol%. The whole-cell fatty acid profile was dominated by C16:0, C17:0 cyclo, and the summed features 3 (C16:1ω7c and/or C16:1ω6c) and 8 (C18:1ω7c and/or C18:1ω6c). Additional fatty acids detected included 12:0, 14:0, and 18:0. Based on these phenotypic, chemotaxonomic, and phylogenetic data, strain JDE115 is proposed to represent a new species in the genus *Pseudomonas*, for which the name '*Candidatus* Pseudomonas auctus' sp. nov. is proposed.

Introduction

The genus *Pseudomonas* was first proposed by Migula in 1894 and is known for its remarkable versatility, metabolic diversity, and environmental ubiquity (1,2). Members of this genus are Gram-negative (3,4), rod-shaped bacteria (5) that are non-spore-forming (6) and motile due to polar flagella (6,7). They are catalase (8,9) and oxidase-positive (10), and their ability to thrive in diverse habitats, including plants, soil, animals, and water, highlights their adaptability and ecological significance (11–16). Currently, more than 200 species of *Pseudomonas* are listed in the bacterial names withstanding in nomenclature (https://www.bacterio.net). Many species within this genus exhibit plant growth-promoting (17–20) and biocontrol (3,4,14,17,21)

properties, making them vital in agriculture for sustainable crop production and disease management. These bacteria contribute to plant health through mechanisms such as phosphate solubilization (22–27), siderophore production (28–32), and the suppression of plant pathogens via direct antagonism or secondary metabolite production (33–36).

However, accurate identification and classification of bacterial species, especially within the diverse genus *Pseudomonas*, require a multifaceted approach (37–40). Among the most robust methods for delineating new species are genomic and phylogenetic analyses (37,41–43). Average Nucleotide Identity (ANI) is widely used for genomic comparisons, with a threshold of 95% often considered indicative of species delineation (44-47). Similarly, digital DNA-DNA Hybridization (dDDH) (48,49), which provides an in silico estimate of DNA-DNA reassociation, has a 70% cutoff for species-level distinctions (48,50). These genome-based metrics complement traditional phylogenetic analyses, such as those based on the 16S rRNA gene, which has been a gold standard for bacterial taxonomy (51–53), and provide additional resolution for closely related taxa (54–58).

Chemotaxonomic approaches further strengthen taxonomic characterization by analyzing cellular fatty acids, respiratory quinones, and polar lipid profiles (59–61). Fatty Acid Methyl Ester (FAME) analysis serves as a complementary or alternative method for detecting soil organisms. It is widely used to characterize microbial community structures and as a diagnostic tool for identifying specific organisms in environmental samples (62). FAME analysis has also been instrumental in differentiating species within *Pseudomonas* (63–66). Specific FAMEs or their ratios can indicate the presence and abundance of certain taxa, serving as biomarkers. For example, the presence of 10:0 3OH, 12:0 3OH, and 14:0 3OH fatty acids indicates a gram-negative organism, while their absence, along with LPS, suggests a gram-positive organism (67).

Respiratory quinones, such as ubiquinone Q-9, and polar lipids, including phosphatidylethanolamine and phosphatidylglycerol, also provide distinctive chemotaxonomic markers (68–71).

Phenotypic and physiological traits play a critical role in the identification and classification of *Pseudomonas* species (72,73). These bacteria are characterized by their Gram-negative cell wall structure (4,19,74), essential for differentiating them from Gram-positive taxa. Their rod-shaped morphology and the presence of one or more polar flagella confer motility, enabling them to colonize diverse environments efficiently (75–77). Non-spore-forming and catalase-positive traits are hallmark features that further delineate members of *Pseudomonas* from other genera (40). Additionally, their oxidase-positive nature reflects their reliance on aerobic respiration, a trait that supports their adaptability in various habitats (10). These phenotypic traits not only confirm their inclusion within *Pseudomonas* but also provide initial clues for distinguishing closely related species. By integrating these phenotypic and physiological attributes with genomic, phylogenetic, and chemotaxonomic analyses, a comprehensive framework for species identification is achieved. Thus, these comprehensive polyphasic approaches are instrumental in accurately determining the taxonomic position of '*Candidatus* Pseudomonas auctus' sp. nov. JDE115.

Materials and methods

Organism and growth condition

'*Candidatus* P. auctus' sp. nov. JDE115 was isolated from surface-sterilized root nodules of soybean (*Glycine max* (L.) Merr.). Briefly, 0.25 g of surface-sterilized soybean nodules (P1)

were homogenized and suspended in 0.9% NaCl solution. The suspension was plated onto King's B medium for bacterial isolation (P2). A single colony of the strain was cultured in LB (Luria–Bertani) lite broth at 28°C for 24 hours under continuous shaking at 200 rpm (P3). Immediately, five samples for glycerol stock (40%) were prepared and stored at -80°C (P4).

Cell morphology and physiological tests

The morphological characteristics of strain JDE115 colonies were assessed following growth on LB lite agar plates. Gram staining was performed according to the protocol described by Bartholomew & Mittwer (1952). Cell morphology and flagellation types were examined using 120 kV FEI T12 Tecnai Spirit transmission electron microscope (TEM) with routine negative staining using Urany-Less® (79) (P5). The attachment of cells to the root surface was observed using a Jeol NeoScope® JCM 5000 scanning electron microscope (SEM) operating at 10kV after sputter coating with 90Å of gold using an Emitech® SC7620 mini sputter coater (80,81). Motility was visualized and recorded using a Leitz® Dialux 22 bright field and phase contrast light microscope equipped with a Nikon® D300 camera to create photomicrographs and digital video (82). Fluorescent pigment production was evaluated by growing JDE115 on King's B medium at 28°C for 24 hours. After incubation, fluorescence was examined using a UV transilluminator (Avantor) under long-wave UV light (365 nm, UV-A) in a darkened environment. Plates were placed directly on the transilluminator surface for visualization (83). Optimal growth conditions for JDE115 were determined by measuring optical density (OD600) in LB liquid cultures (84,85). Growth was evaluated at temperatures ranging from 4°C to 40°C in 4°C increments over 24 hours and at pH levels from 4.0 to 10.0 to determine the strain's tolerance and preferences (2,74,86).

Physiological assessments, including salt tolerance, pH range, temperature range, motility, and aerobic metabolism, alongside comprehensive biochemical tests (unpublished, Ali et al.), were conducted to characterize '*Candidatus* Pseudomonas auctus' nov. sp. JDE115. Cellular fatty acid profiles were analyzed using the Sherlock 6.1 system (Microbial Identification Inc.) with the RTSBA6 library (67), providing detailed insights into the strain's fatty acids composition (P6). Biochemical properties and enzymatic activities were evaluated using Biolog® GENIII Microplates (87–90) (P7). Results were recorded after 48 hours of incubation at 28°C, yielding a robust dataset highlighting the metabolic versatility and adaptive features of JDE115.

Phylogenetic analysis

Bacteria from glycerol stock were streaked onto Luria-Bertani agar (LBA) plates and incubated to obtain isolated colonies. A single colony was transferred into 50 mL of LB lite broth (containing 5 g/L yeast extract and 10 g/L NaCl) and incubated at 28°C for 16 hours with continuous shaking at 200 rpm. The culture reached an optical density (OD) of approximately 0.6. From this actively growing culture, 1.8 mL was used for genomic DNA extraction using the DNeasy UltraClean Microbial Kit (Qiagen®, USA) (P8). The extracted DNA was quantified using a NanoDrop 1000 spectrophotometer and a Qubit 3.0 fluorometer (Thermo Fisher Scientific®, USA). DNA purity was further assessed by agarose gel electrophoresis (P9). A nearly full-length 16S rRNA gene was amplified using the universal primers 27F (5′-AGAGTTTGATCMTGGCTCAG-3′) and 1492R (5′-TACGGHTACCTTGTTACGACTT-3′) through PCR (74,91,92). PCR was performed using a Bio-Rad® Thermal Cycler, and the products were purified with a QIAquick® PCR Purification Kit (QIAGEN®) (P10). Sanger sequencing reactions were carried out by the ABI 3730XL.

Computation of the phylogenetic tree based on 16S rRNA was performed using SeaView® (93,94) with the maximum likelihood approach with the 1000 bootstrap replicates (95,96). Sequences of type strains used in the phylogenetic analysis were downloaded from NCBI (accession numbers in Table 3).

The genomic DNA was sheared into fragments using a Covaris® S220 ultrasonicator, and sequencing libraries were prepared using the Illumina-compatible NEBNext Ultra II DNA Library Prep Kit (New England BioLabs®, USA). Sequencing was performed on the Illumina® NovaSeq PE150 platform at the NGS core facility, generating paired-end reads of 150 bp. FASTQ files were generated using Guppy (v4.4.1), and sequences with a Phred score below 7 were excluded. De novo assembly was performed using Flye (v2.9) (97), with default parameters and a genome size setting of 6.2 Mbp. Assembly completeness was confirmed using dot plots generated with Gepard (98). The complete genome was visualized using CGView (99,100). Genome annotation was conducted using the NCBI Prokaryotic Genome Annotation Pipeline (PGAP) and the RAST server (101). The Whole Genome Shotgun sequence project has been deposited in GenBank under accession number JBJYZK000000000.

Similarity analyses (ANI and GGDC) of the sequenced genome of strain JDE115 to other 40 genomes of the closely related *Pseudomonas* species were determined as briefed below. ANI based on pairwise comparison was calculated using the software FastANI (44). GGDC was calculated using the web service http://ggdc.dsmz.de and using the recommended BLAST+method (49). The GGDC results shown are based on the recommended formula 2 (sum of all identities found in HSPs divided by the overall HSP length), which is independent of the genome length and is thus robust against the use of incomplete draft genomes.

The Type (Strain) Genome Server (TYGS, https://tygs.dsmz.de) was employed for whole-genome-based taxonomic analysis, incorporating recent methodological updates (102,103). TYGS identified closely related type strains through two complementary approaches: (1) comparison of user genomes with type strain genomes in the TYGS database using the MASH algorithm (104) to identify the ten closest type strains and (2) extraction of 16S rDNA sequences from user genomes using RNAmmer (105), followed by BLAST (106) analysis against 16S rDNA gene sequences from 22,195 type strains in the TYGS database. These methods were used to determine the closest type strain genomes based on intergenomic distances calculated using the Genome Blast Distance Phylogeny (GBDP) approach with the “coverage” algorithm and distance formula d_5 (49). Pairwise genome comparisons were performed with GBDP to infer intergenomic distances and calculate digital DNA-DNA hybridization (dDDH) values using GGDC 4.0. Phylogenomic analysis was conducted by constructing a balanced minimum evolution tree using FASTME 2.1.6.1 with 100 pseudo-bootstrap replicates for branch support (107). The tree was rooted at the midpoint (108) and visualized using PhyD3 (109). Species clustering was based on a 70% dDDH threshold (103), while subspecies clustering used a 79% dDDH threshold, as previously described (110). These analyses provided insights into the taxonomic positioning of the studied genome. Taxonomic and nomenclatural information was also supplemented by the List of Prokaryotic Names with Standing in Nomenclature (LPSN, https://lpsn.dsmz.de) (102).

Chemotaxonomic analysis

The cellular fatty acid profile of JDE115, a critical chemical characteristic for bacterial identification, was analyzed to support its taxonomic classification. Fatty acids were harvested

after 24 hours of incubation at 28°C on Tryptic Soy Agar (TSA). The extraction process involved saponification, methylation, and subsequent extraction following the standardized protocols of the MIDI System (Sherlock Microbial Identification System, version 6.0B). Analysis was performed using gas chromatography (GC; 6850, Agilent Technologies®), and the fatty acids were identified utilizing the TSBA6.0 database of the Microbial Identification System (67).

Results and discussion

Phenotype analysis

‘*Candidatus* P. auctus’ sp. nov. JDE115 was observed to be gram-negative, rod-shaped (µm) (Fig 1), fluorescent (S1, Fig), aerobic or facultatively anaerobic, motile (S1, movie) with monotrichous flagella (Fig 1). Colonies grown on LB at 28°C for 24 h are light yellow, round, smooth, convex, waxy and translucent, 4.0–5.0 mm in diameter (S2, Fig). Growth occurs in 0–4% (w/v) NaCl (optimum 1%), at pH 4.0–10.0 (optimum pH 7.0), and at 10–40°C (optimum 28 °C). Fluorescent pigments were observed when cultured for 24 hr at 28°C on King’s B medium (S1, Fig). The physiological, morphological, and phenotypic characteristics in Biolog GEN III tests, which showed differentiation of strains JDE115 from other closely related *Pseudomonas* species, are listed in Table 1.

Fig 1. Transmission electron micrograph of ‘*Candidatus* Pseudomonas auctus’ nov. sp. JDE115. This rod-shaped bacterium has a monotrichous polar flagellum that is more than twice as long as its body.

Table 1. Differentiating characteristics of '*Candidatus* Pseudomonas auctus' sp. nov. strain JDE115 from other related species of *Pseudomonas*.

Characteristics	***P. auctus* JDE115**	***P. glycinae* MS586^{T}**	***P. kribbensis* 46-2^{T}**	***P. granadensis* F-278**	***P. moraviensis* 1B4^{T}**	***P. koreensis* Ps9-14^{T}**	***P.baetica* a390T**	***P. vancouverensis* DhA-51^{T}**	***P. jessenii* DSM 17150^{T}**	***P. reinekei* MT1^{T}**
Flagellation	Polar, Single	Polar, multiple	Polar, multiple	Polar, two	Polar, two	Polar, multiple	ND	ND	Polar, single	ND
Fluorescence										
Growth at 4°C								ND[a]		ND
Tolerance of NaCl at 5%										
Nitrate reduction										
Arginine dihydrolase										
Hydrolysis of gelatin										
Citrate utilization										
Urease						ND				
Assimilation of l-Arabinose										
N-Acetyl-d-glucosamine										
Phenylacetic acid										
d-Mannose										
Dextrin										

Tween-40										
d-Cellobiose										
d-Trehalose										
l-Arabinose										
d-Fructose								ND		
d-Mannitol										
d-Arabitol										
l-Alanine										
l-Serine										
α-Ketobutyric acid										
α-Ketoglutaric acid										
Glucuronamide										
l-Histidine										
d-Serine										
d-Galactose										
d-Galacturonic acid			ND			ND				
d-Glucuronic acid										
Glucuronamide					ND					
p-Hydroxy phenylacetic acid										
Quinic acid										
d-Saccharic acid										
Glycyl-l-proline			ND							
L-Pyroglutamic acid						ND				
Inosine										

Propionic acid										
Formic acid										
Acetic acid										
Methyl pyruvate										
GC content (%)	60.7	60.5	60.5	59.9	60.3	59.1	58.7	67.2	62.2	59.1

Comparison of key phenotypic and biochemical characteristics of '*Candidatus* Pseudomonas auctus' nov. sp. JDE115 with closely related *Pseudomonas* species, including flagellation type, fluorescence, growth at 4°C, NaCl tolerance, nitrate reduction, and various metabolic and enzymatic activities. Biochemical reactions were assessed using the Biolog GEN III system, which evaluates the utilization of 71 carbon sources and the resistance to 23 chemical agents for comprehensive bacterial identification. Data for strain '*Candidatus* Pseudomonas auctus' nov. sp. JDE115 were obtained in this study. Data for other type strains were obtained from references, *P. glycinae* MS586^{T} Jia et al. 2020 (74); *P. kribbensis* 46-2^{T} Chang et al. 2016 (111); *P. granadensis* F-278,770^{T} Pascual et al. 2015 (112); *P. moraviensis* 1B4^{T} Pascual et al. 2015 (112); *P. koreensis* Ps9-14^{T} Tvrzová et al. 2006 (113); *P.baetica* a390^{T} López et al. 2012 (114); *P. vancouverensis* DhA-51^{T} Cámara et al. 2007 (115); *P. jessenii* DSM 17150^{T} Cámara et al. 2007 (115); *P. reinekei* MT1^{T} Cámara et al. 2007 (115).

Abbreviations: dark green is for positive reaction; red is for negative reaction; light green is for weak reaction; and ND[a] is for no data.

Phylogenetic analysis

Sequence analysis of the 16S rRNA gene revealed that '*Candidatus* P. auctus' nov. sp. JDE115 shares significant sequence identity (>99%) with several *Pseudomonas* species. The

closely related strains include *P. glycinae* MS586^T (99.86%), *P. kribbensis* 46-2^T (99.71%), *P. soyarea* JJL17^T (99.36%), *P. koreensis* Ps 9-14^T (99.36%), *P. reinekei* MT1^T (99.29%), and *P. moraviensis* 1B4^T (99.29%). Due to the high sequence similarity, the 16S rRNA gene alone cannot determine the precise taxonomic position of these closely related species (116,117). This limitation arises from the high conservation of 16S rRNA variable regions, taxon-specific evolutionary constraints, and detection biases, which often hinder its ability to resolve within-genus taxonomy accurately (118). Recent studies have also highlighted that evolutionary rigidity and horizontal gene transfer associated with the 16S rRNA gene contribute to insufficient diversification among closely related taxa, resulting in limited resolution at the species level (119). Further whole-genome analyses and phenotypic characterizations are required to clarify the taxonomic placement and distinguish '*Candidatus* P. auctus' nov. sp. JDE115 from its closely related species.

However, A phylogenetic tree based on the 16S rRNA gene sequences was constructed to assess the evolutionary relationship of '*Candidatus* P. auctus' nov. sp. JDE115 with other closely related species. The analysis revealed that JDE115 shares a high sequence similarity with multiple *Pseudomonas* species, including *P. glycinae* MS586^T, *P. kribbensis* 46-2^T, and *P. soyarea* JJL17^T, forming a distinct cluster within the *Pseudomonas* genus. Despite the close relationships, JDE115 formed a unique branch with high bootstrap support, indicating its distinct taxonomic position (Fig 2).

Fig 2. Phylogenetic tree illustrating the evolutionary relationship of '*Candidatus* Pseudomonas auctus' nov. sp. JDE115 with closely related species based on 16S rRNA gene sequences. The tree was created using the Maximum Likelihood method through the PhyML engine integrated into SeaView software (Gouy et al., 2010). Bootstrap values above 50% are

shown at the branch nodes, supporting the branching patterns. This analysis highlights the high sequence similarity between JDE115 and other species within the genus *Pseudomonas* while demonstrating the distinct taxonomic position of JDE115 through a unique branch with significant bootstrap support (highlighted in green).

General genome features

The genome of ‘*Candidatus* Pseudomonas auctus’ nov. sp. JDE115 was fully sequenced and annotated, revealing a genome size of 6,183,199 base pairs (bp) with a GC content of 60.68%, the genome size of *Pseudomonas* typically range from 4.2 to 7Mba (120,121). Besides, the GC content of JDE115 falls within the reported range of 48-68 mol% for members of the genus *Pseudomonas* (122–126). The genome comprises 5,648 genes, of which 5,509 (97.54%) are coding genes and 70 (1.24%) are RNA genes. Additionally, the genome contains 69 pseudogenes (1.22%), representing a minor fraction of the total genetic content. The coding DNA sequence (CDS) accounted for 5,578 genes, contributing to 98.76% of total genes (Table 2). The number of coding sequences (CDS) within the genus of *Pseudomonas* exhibits substantial variability, ranging from 4,274 to 6,305 (127).

Table 2. General taxonomic genome features of ‘*Candidatus* Pseudomonas auctus’ nov. sp. JDE115.

**Attributes**	**Value**	**% of total**
Genome size (bp)	6,183,199	100
DNA Coding (bp)	5,513,388	89.17
GC Content (%)	3,752,162	60.68

Genes (total)	5,648	100
CDS (total)	5,578	98.76
Genes (coding)	5,509	97.54
Genes (RNA)	70	1.24
Pseudo Genes	69	1.22

Summary of the genome assembly and annotation of ‘*Candidatus* Pseudomonas auctus’ nov. sp. JDE115, including genome size, GC content, total gene count, coding sequences (CDS), RNA genes, and pseudogenes.

The ANI analysis revealed the highest similarity of ‘*Candidatus* P. auctus’ nov. sp. JDE115 with *P. glycinae* MS586, with a value of 94.59%, followed by *P. fitomatiaceae* FIT81 (94.24%) and *P. guzikowskii* IzPS32d (93.92%). However, these values fall below the commonly accepted ANI threshold of 95% for species delineation (44). ANI comparisons between genomes and type strain assemblies have corrected approximately 750 previously misidentified entries in GenBank, underscoring its critical role in taxonomic accuracy (128). Additionally, ANI's ability to handle large datasets with high computational efficiency makes it ideal for modern microbial taxonomy, supporting robust species-level classifications even in complex datasets (129). Similarly, GGDC values ranged from 57.10% with *P. glycinae* MS586 to as low as 21.30% (*P. wenzhouensis* A20) with more distantly related species (Table 3).

The comparative genomic analysis underscored the unique genetic identity of ‘*Candidatus* P. auctus’ nov. sp. JDE115. While ANI and GGDC analyses identified significant genomic similarities with certain *Pseudomonas* species, the values consistently remained below the established cutoffs (95% for ANI and 70% for GGDC) for species-level classification (130).

Notably, JDE115 shared substantial genomic relatedness with species like *P. glycinae* MS586 (94.59 and 57.10), *P. fitomaticsae* FIT81 (94.24 and 54.80), *P. gozinkensis* IzPS32d (93.92 and 54.00), *P. kribbensis* 46-2 (91.46% ANI and 42.50% dDDH) and *P. alokibbiensis* IzPS23 (91.74% ANI and 43.00% GGDC), yet the data unequivocally supports the novelty of JDE115 as a distinct taxonomic entity.

Table 3. ANI (%) and GGDC (%) between '*Candidatus* Pseudomonas auctus' nov. sp. JDE115 and closely related sequenced strains of the genus *Pseudomonas.* Average

***Pseudomonas* species**	**Genome accession number**	**ANI (%)**	**DDH (%)**
P. glycinae MS586	GCF_001594225.2	94.59	57.10
P. fitomaticsae FIT81	GCF_021018765.1	94.24	54.80
P. gozinkensis IzPS32d	GCF_014863585.1	93.92	54.00
P. allokribbensis IzPS23	GCF_014863605.1	91.74	43.00
P. kribbensis 46-2	GCF_003352185.1	91.46	42.50
P. monsensis PGSB 8459	GCF_014268495.2	88.43	33.60
P. zeae OE 48.2	GCF_014268485.2	88.20	33.00
P. tensinigenes ZA 5.3	GCF_014268445.2	88.12	32.90
P. germanica FIT28	GCF_019614655.1	88.07	32.70
P. iranensis SWRI54	GCF_014268585.2	87.67	32.10
P. hamedanensis SWRI165	GCF_014268595.2	87.32	31.70
P. grandcourensis DGS24	GCF_039909015.1	86.53	29.60
P. hormoni G20-18	GCF_018502625.1	86.41	29.60

P. azerbaijanorientalis SWRI123	GCF_019139795.1	86.41	29.90
P. nunensis ln5	GCF_024296925.1	86.29	29.30
P. wuhanensis FP607	GCF_030687395.1	86.15	29.30
P. svalbardensis PMCC 200367	GCF_030053115.1	86.10	29.00
P. cucumis FP1935	GCF_030687935.1	86.07	28.90
P. silesiensis	GCF_001661075.1	85.76	29.30
P. purpurea DGS26	GCF_039908635.1	85.30	27.20
P. chlororaphis ATCC 9446	GCF_036689615.1	85.12	26.80
P. helvetica DGS28	GCF_039908645.1	85.01	27.00
P. ogarae SWRI108	GCF_014268695.2	84.60	26.90
P. viciae 11K1	GCF_004786035.1	84.53	26.50
P. alvandae SWRI17	GCF_019141525.1	84.29	26.60
P. protegens CHA0	GCF_900560965.1	84.16	25.40
P. hefeiensis FP205	GCF_030687835.1	84.03	26.10
P. shahriarae SWRI52	GCF_014268455.2	83.69	25.10
P. pergaminensis 1008	GCF_024112395.2	83.64	24.90
P. asgharzadehiana SWRI132	GCF_019139815.1	83.53	24.60
P. tritici SWRI145	GCF_014268275.3	83.42	24.50
P. salmasensis SWRI126	GCF_014268375.2	83.33	24.80
P. vanderleydeniana RW8P3	GCF_014268755.2	83.30	24.80
P. sessilinigenes CMR12a	GCF_003850565.1	83.07	24.30
P. mucidolens NCTC8068	GCF_900475945.1	82.91	24.40
P. tructae SNU WT1	GCF_004214895.1	82.11	22.90

P. abieticivorans PIA16	GCF_023509015.1	81.89	23.10
P. oryziphila 1257	GCF_003940825.1	81.76	22.70
P. muyukensis COW39	GCF_019139535.1	81.73	22.40
P. eucalypticola NP-1	GCF_013374995.1	81.63	22.80
P. fakonensis COW40	GCF_019139895.1	81.63	22.40
P. versuta L10.10	GCF_001294575.1	81.62	23.30
P. maumuensis COW77	GCF_019139675.1	81.58	22.10
P. xantholysinigenes RW9S1A	GCF_014268885.2	81.56	22.40
P. xanthosomatis COR54	GCF_019139835.1	81.56	22.60
P. entomophila L48	GCF_000026105.1	81.52	22.40
P. lijiangensis LJ2	GCF_018968705.1	81.44	22.60
P. taetrolens NCTC10697	GCF_900475285.1	81.34	23.10
P. cichorii DSM 50259	GCF_018343775.1	81.31	22.60
P. anuradhapurensis RD8MR3	GCF_014269225.2	81.24	22.70
P. rhizosphaerae DSM 16299	GCF_000761155.1	81.24	22.80
P. putida NBRC 14164	GCF_000412675.1	81.24	22.20
P. fortuita GMI12077	GCF_026898135.2	81.05	22.10
P. syringae pv. tomato str. DC3000	GCF_000007805.1	80.87	22.50
P. cavernae K2W31S-8	GCF_003595175.1	80.72	21.90
P. huanghezhanensis BSw22131	GCF_026810445.1	80.40	22.00
P. promysalinigenes RW10S1	GCF_014269025.2	80.36	21.60
P. syringae pv. *Tagetis* ICMP 4091	GCF_022557255.1	80.32	22.00
P. tohonis TUM18999	GCF_012767755.2	80.18	21.30

P. wenzhouensis A20	GCF_021029445.1	80.10	21.60

Nucleotide Identity (ANI) and Genome-to-Genome Distance Calculator (GGDC) values comparing ‘*Candidatus* Pseudomonas auctus’ nov. sp. JDE115 with closely related *Pseudomonas* species. ANI values above 95% typically indicate species-level relatedness, while GGDC values above 70% suggest the same species.

A whole-genome sequence-based phylogenetic analysis was conducted for ‘*Candidatus* P. auctus’ nov. sp. JDE115 and closely related species within the genus *Pseudomonas*. The tree was inferred using the Genome BLAST Distance Phylogeny (GBDP) approach implemented in the TYGS platform. Distances were calculated from genome sequences using formula d5. The resulting phylogenetic tree illustrated that JDE115 formed a distinct branch within the genus, clustering most closely with *P. glycinae* MS586 and *P. kribbensis* KCTC 32541^T. Branch lengths in the tree are scaled in terms of GBDP distance formula d5, and bootstrap values from 100 replicates are provided at each node, indicating strong statistical support for the inferred relationships. High bootstrap support values (≥90) were observed, particularly in the branching patterns distinguishing ‘*Candidatus* P. auctus’ nov. sp. JDE115 from its closest relatives, reinforcing its phylogenetic distinctiveness (Fig 3). The closest relatives of JDE115 in the phylogenetic tree include *P. glycinae* MS586 (TYGS ID: 37260), *P. kribbensis* KCTC 32541^T (TYGS ID: 14671), and *P. moraviensis* LMG 24280 (TYGS ID: 19005). The accession numbers and strain names of all related species are summarized in Table 4. These data underscore the unique taxonomic position of JDE115 within the genus *Pseudomonas* based on whole-genome analysis. Having established the genomic distinctiveness of JDE115, its chemotaxonomic characteristics were studied to further validate its taxonomic classification.

Fig 3. Whole-genome sequence tree generated with TYGS for '*Candidatus* Pseudomonas auctus' nov. sp. JDE115 and its closely related species of the genus *Pseudomonas*. Tree inferred with FastME from GBDP distances was calculated from genome sequences. Branch lengths are scaled in terms of GBDP distance formula d5; numbers above branches are GBDP pseudo-bootstrap support values from 100 replications. Accession numbers of sequences used in this study are summarized in Table 4.

Table 4. Accession numbers for genome sequences of various *Pseudomonas* species strains utilized in whole-genome phylogenetic analysis.

Species	Biosample accession	Strains name	TYGS ID	Isolation Source	Authority
P. bananamidigenes	SAMN04444410	BW11P2	100258	Rhizosphere	(123)
P. aphyarum	SAMN28093775	ID386	104585	Aquatic environments	(125)
P. fitomaticsae	SAMN19241968	FIT81	109195	Rhizosphere	(122)
P. kribbensis	SAMN09244908	32541^{T}	14671	Soil or environmental	(111)
P. iranensis	SAMN15248341	SWRI54	147537	Rhizosphere	(123)
P. ekonensis	SAMN19473669	COR58	147556	Rhizosphere	(123)
P. moraviensis	SAMN04490196	LMG 24280	19005	**Unknown**	(113)
P. atacamensis	SAMN11356701	M7D1	22501	Rhizosphere	(117)
P. glycinae	SAMN04435621	MS586	37260	Rhizosphere	(74)
P. koreensis	SAMD00245249	JCM 14769	48496	Soil or environmental	(131)
P. botevensis	SAMN19473668	COW3	82604	Rhizosphere	(123)
P. serboccidentalis	SAMN29793685	IT-P374^{T}	86200	Rhizosphere	(126)
P. gozinkensis	SAMN16250066	LMG 31526	92631	Soil or environmental	(132)
P. allokribbensis	SAMN16250065	LMG31525^{T}	92633	Soil or environmental	(132)

'*Candidatus* Pseudomonas auctus' nov. sp.	SAMN45708326	JDE115	U884796	Rhizosphere	This study

This table provides the genome accession numbers, strain names, TYGS ID, and isolation sources of *Pseudomonas* species used in the whole-genome sequence-based phylogenetic analysis. '*Candidatus* Pseudomonas auctus' nov. sp. JDE115 was sequenced in this study

Chemotaxonomic analysis

Cellular fatty acids were analyzed for '*Candidatus* P. auctus' nov. sp. JDE115 using the Sherlock 6.1 system (Microbial Identification Inc.) and the RTSBA6 library (67). The dominant fatty acids detected for JDE115 included C16:0 (25.07%), summed feature 3 (C16:1ω7c/C16:1ω6c) (34.78%), summed feature 8 (C18:1ω7c/C18:1ω6c) (16.91%), and C17:0 cyclo (0.87%). Additionally, fatty acids C12:0 (1.88%), C14:0 (0.66%), and C18:0 (1.35%) was identified (Table 5). These profiles are consistent with those typically observed in the genus *Pseudomonas*. The presence of three characteristic fatty acids—C10:0 3-OH, C12:0, and C12:0 3-OH—further confirms the taxonomic affiliation of JDE115 within *Pseudomonas*.

Table 5. Distinct cellular fatty acid profiles of strain '*Candidatus* Pseudomonas auctus' nov. sp. JDE115 compared with related *Pseudomonas* strains.

Fatty acid	*P. auctus* JDE115	*P. glycinae* MS586^T	*P. kribbensis* 46-2^T	*P. granadensis* F-278	*P. moraviensis* 1B4^T	*P. koreensis* Ps9-14^T	*P.baetica* a390T	*P. vancouverensis* DhA-51^T	*P. jessenii* DSM 17150^T	*P. reinekei* MT1^T
C10:0 3-OH	5.5	6.6	5.4	3.2	2.6	2.2	3.4	4.8	2.8	3.3
C12:0 2-OH	6.38	5.5	6.8	4.7	4.9	5	5.5	3.8	5.5	4.3
C12:0 3-OH	5.62	6.7	7.5	2.5	4.1	4	3.2	5.7	3.2	4.8
C10:0	ND	0.8	ND	ND	ND	ND	0.1	0.3	0.1	ND
C12:0	1.88	2.9	ND	1.5	2.1	1.6	1.7	3.8	4.7	3.6
C14:0	0.66	0.6	1.2	ND	0.4	0.7	0.5	0.6	0.3	0.7
C16:0	25.07	22.9	33.4	32	29	33	29.4	29.4	29.4	36.5
C17:0 cyclo	0.87	10.3	15.1	6.9	2.4	2	3.2	9.4	0.9	22.3
C18:0	1.35	0.3	1.6	ND	0.5	0.7	0.3	0.2	0.7	0.8
C19:0 ω8c	ND	1.2	ND	ND	0.2	ND	ND	ND	ND	0.7
Summed feature 3	34.78	23.6	16.8	36	36	37	39.5	30.8	38.1	28
Summed feature 8	16.91	13.4	8.9	12	17	13	12.2	8.5	17.2	8.6

Fatty acid composition of '*Candidatus* Pseudomonas auctus' nov. sp. JDE115 and closely related *Pseudomonas* species, analyzed using the Sherlock 6.1 system (Microbial Identification Inc.) and the RTSBA6 library. The values represent the percentage of total fatty acids detected. Summed features refer to groups of two or three fatty acids that cannot be individually resolved using gas chromatography (GC) with the MIDI system. Summed feature 3 includes C16:1ω7c and/or C16:1ω6c, while summed feature 8 comprises C18:1ω7c and/or C18:1ω6c. Data for strain

‘*Candidatus* Pseudomonas auctus’ nov. sp. JDE115 were obtained in this study. Data for other type strains were obtained from references, *P. glycinae* MS586^{T} (74); *P. kribbensis* 46-2^{T} (111); *P. granadensis* F-278,770^{T} (112); *P. moraviensis* 1B4^{T} (112); *P. koreensis* Ps9-14^{T} (113); *P.baetica* a390^{T} (114); *P. vancouverensis* DhA-51^{T} (115); *P. jessenii* DSM 17150^{T} (115); *P. reinekei* MT1^{T} (115).

Abbreviation: ND indicates fatty acids that were not detected.

Compared to closely related species, such as *P. glycinae* MS586T, which shows C16:0 at 22.9%, summed feature 3 at 23.57%, summed feature 8 at 13.37%, and C17:0 cyclo at 10.28%, JDE115 exhibits a higher proportion of summed features 3 and 8 but lower C17:0 cyclo. These differences emphasize the unique chemotaxonomic profile of JDE115 within the genus. Moreover, the characteristic fatty acids C10:0 3-OH, C12:0, and C12:0 3-OH, commonly observed in *Pseudomonas* species, were detected in ‘*Candidatus* P. auctus’ nov. sp. JDE115, further validating its classification within the genus.

Strains like *P. kribbensis* 46-2^{T} and *Pseudomonas moraviensis* 1B4^{T} exhibit variations in their fatty acid profiles. For instance, *P. kribbensis* 46-2^{T} has a significantly higher proportion of C16:0 (33.4%) and C17:0 cyclo (15.1%) than JDE115, while *P. moraviensis* 1B4^{T} shows C16:0 at 32% and summed feature 3 at 36%, reflecting its distinct metabolic adaptations (Table 5). The chemotaxonomic differentiation is further highlighted by unique percentages in summed feature 8 and the presence of minor fatty acids like C19:0 ω8c in JDE115, which were not detected in many other *Pseudomonas* strains. These comparative findings underscore the novelty of ‘*Candidatus* P. auctus’ nov. sp. JDE115's chemotaxonomic profile and its distinct position within the genus.

Conclusions

Based on a comprehensive polyphasic approach integrating phenotypic, molecular, and chemotaxonomic analyses, '*Candidatus* P. auctus' nov. sp. JDE115 is proposed as a novel species within the genus *Pseudomonas*. Its distinct taxonomic position is strongly supported by divergence in genomic similarity indices, including ANI and GGDC, which fall below the species delineation thresholds compared to its closest relatives. Phenotypic characteristics, such as motility, Gram-negative and rod-shaped morphology, facultative aerobiosis, and catalase- and oxidase-positive activity, align with traits typical of the genus while demonstrating unique growth conditions and fluorescence properties. Additionally, the chemotaxonomic profile, including dominant fatty acids such as C16:0, summed feature 3 (C16:1ω7c/C16:1ω6c), and summed feature 8 (C18:1ω7c/C18:1ω6c), further confirms its placement within *Pseudomonas* while distinguishing it from closely related species. These findings underscore the ecological and taxonomic significance of JDE115, providing a strong foundation for exploring its potential in plant growth promotion and biocontrol. The name '*Candidatus* P. auctus' nov. sp. is proposed. It remains *candidatus* because the type strain cannot be released to the public because of its potential development as a biopesticide.

Description of '*Candidatus* Pseudomonas auctus' nov. sp.

'*Candidatus* P. auctus' nov. sp. (auc'tus. L. masc. adj. auctus, meaning growth or increase, referring to its ability to promote plant growth) is an aerobic or facultative anaerobic, gram-negative, rod-shaped bacterium with motility conferred by polar flagella. Colonies are

fluorescent and appear light-yellow when grown on King's B agar plates. On LB agar, colonies are smooth, circular, slightly convex, and 5.0–7.5 mm in diameter after 72 hours of incubation at 28°C. Cells are approximately 0.7-.09 X 2.2-3.0 μm in size. Growth occurs at temperatures ranging from 4°C to 40°C, with an optimum temperature of 28–30°C. The bacterium can grow within a pH range of 4.0–10.0, with an optimum pH of 6.0–7.0. '*Candidatus* P. auctus' nov. sp. tolerates salinity up to 4% (w/v) NaCl, with optimal growth observed at 1% NaCl.

Biochemical and metabolic characterization using Biolog GENIII Micro Plates (S1, Table) demonstrated the ability of '*Candidatus* P. auctus' nov. sp. JDE115 to utilize a variety of substrates. The strain exhibited positive utilization for several carbohydrates, including α-d-glucose, d-mannose, d-fructose, d-galactose, d-fucose, l-fucose, l-rhamnose, and d-mannitol. Amino acid utilization included d-aspartic acid, l-aspartic acid, l-glutamic acid, l-histidine, l-alanine, l-arginine, l-serine, and glycyl-l-proline. The strain also metabolized organic acids such as citric acid, l-malic acid, α-ketoglutaric acid, γ-aminobutyric acid, d-saccharic acid, and acetic acid. '*Candidatus* P. auctus' nov. sp. JDE115 demonstrated growth in the presence of chemical inhibitors such as fusidic acid, troleandomycin, rifamycin SV, vancomycin, guanidine HCl, and potassium tellurite, reflecting its adaptive tolerance to diverse chemical conditions. It also exhibited positive results for pH 6 and 5 and tolerated NaCl concentrations up to 4%, but growth was inhibited at 8%. The strain was resistant to aztreonam, nalidixic acid, and lithium chloride but was inhibited by sodium butyrate.

Phylogenetic analysis based on 16S rRNA gene sequences revealed that '*Candidatus* P. auctus' nov. sp. JDE115 shares high sequence similarity (>99%) with *P. glycinae* MS586^T, *P. kribbensis* 46-2^T, and *P. soyarea* JJL17^T. However, whole-genome-based ANI (94.59%) and GGDC (57.10%) values with its closest relative, *P. glycinae* MS586^T, fall below the thresholds

for species delineation, confirming its status as a novel species. The strain JDE115 (=SAMN45708326), isolated from soybean root nodules at Kentland Farm, McCoy, Virginia, USA.

Data availability

Genome data has been deposited in GenBank under accession number JBJYZK000000000.

Conflict of interest

The authors declare no conflicts of interest.

Funding statement

This research was supported by the Virginia Tech Intellectual Property LAUNCH Proof-of-Concept grant and the School of Plant and Environmental Sciences.

Acknowledgments

The authors would like to acknowledge Virginia Tech Intellectual Property for supporting this research and extend our gratitude to the School of Plant and Environmental Sciences at Virginia Tech for providing the resources and facilities necessary to conduct this study.

References

1. Migula, W. Über ein neues System der Bakterien. Arb Bakteriol Inst Karlsruhe. 1894;1:235–8.

2. Yang G, Han L, Wen J, Zhou S. *Pseudomonas guangdongensis* sp. nov., isolated from an electroactive biofilm, and emended description of the genus *Pseudomonas* Migula 1894. Int J Syst Evol Microbiol. 2013 Dec 1;63(Pt_12):4599–605.

3. Albert D, Zboralski A, Ciotola M, Cadieux M, Biessy A, Blom J, et al. Identification and genomic characterization of *Pseudomonas* spp. displaying biocontrol activity against *Sclerotinia sclerotiorum* in lettuce. Front Microbiol. 2024 Mar 7;15:1304682.

4. Wang X, Zhou X, Cai Z, Guo L, Chen X, Chen X, et al. A Biocontrol Strain of *Pseudomonas aeruginosa* CQ-40 Promote Growth and Control *Botrytis cinerea* in Tomato. Pathogens. 2020 Dec 31;10(1):22.

5. Duvernoy MC, Mora T, Ardré M, Croquette V, Bensimon D, Quilliet C, et al. Asymmetric adhesion of rod-shaped bacteria controls microcolony morphogenesis. Nat Commun. 2018 Mar 16;9(1):1120.

6. Liu Y, Song Z, Zeng H, Lu M, Zhu W, Wang X, et al. *Pseudomonas eucalypticola* sp. nov., a producer of antifungal agents isolated from *Eucalyptus dunnii* leaves. Sci Rep. 2021 Feb 4;11(1):3006.

7. Krieg NR, Staley JT, Brown DR, Hedlund BP, Paster BJ, Ward NL, et al., editors. Bergey's Manual® of Systematic Bacteriology: Volume Four The Bacteroidetes, Spirochaetes, Tenericutes (Mollicutes), Acidobacteria, Fibrobacteres, Fusobacteria, Dictyoglomi, Gemmatimonadetes, Lentisphaerae, Verrucomicrobia, Chlamydiae, and Planctomycetes [Internet]. New York, NY: Springer New York; 2010 [cited 2025 Jan 22]. Available from: https://link.springer.com/10.1007/978-0-387-68572-4

8. Shin DH, Choi YS, Cho YH. Unusual Properties of Catalase A (*KatA*) of *Pseudomonas aeruginosa* PA14 Are Associated with Its Biofilm Peroxide Resistance. J Bacteriol. 2008 Apr 15;190(8):2663–70.
9. Su S, Panmanee W, Wilson JJ, Mahtani HK, Li Q, VanderWielen BD, et al. Catalase (KatA) Plays a Role in Protection against Anaerobic Nitric Oxide in *Pseudomonas aeruginosa*. Roop RM, editor. PLoS ONE. 2014 Mar 24;9(3):e91813.
10. Da Cruz Nizer WS, Inkovskiy V, Versey Z, Strempel N, Cassol E, Overhage J. Oxidative Stress Response in *Pseudomonas aeruginosa*. Pathogens. 2021 Sep 14;10(9):1187.
11. Frimmersdorf E, Horatzek S, Pelnikevich A, Wiehlmann L, Schomburg D. How *Pseudomonas aeruginosa* adapts to various environments: a metabolomic approach. Environ Microbiol. 2010 Jun;12(6):1734–47.
12. Kordes A, Preusse M, Willger SD, Braubach P, Jonigk D, Haverich A, et al. Genetically diverse *Pseudomonas aeruginosa* populations display similar transcriptomic profiles in a cystic fibrosis explanted lung. Nat Commun. 2019 Jul 30;10(1):3397.
13. Mei S, Wang M, Salles JF, Hackl T. Diverse rhizosphere-associated *Pseudomonas* genomes from along a Wadden Island salt marsh transition zone. Sci Data. 2024 Oct 17;11(1):1140.
14. Shalev O, Karasov TL, Lundberg DS, Ashkenazy H, Pramoj Na Ayutthaya P, Weigel D. Commensal *Pseudomonas* strains facilitate protective response against pathogens in the host plant. Nat Ecol Evol. 2022 Feb 24;6(4):383–96.
15. Silby MW, Winstanley C, Godfrey SAC, Levy SB, Jackson RW. *Pseudomonas* genomes: diverse and adaptable. FEMS Microbiol Rev. 2011 Jul;35(4):652–80.

16. Thöming JG, Tomasch J, Preusse M, Koska M, Grahl N, Pohl S, et al. Parallel evolutionary paths to produce more than one *Pseudomonas aeruginosa* biofilm phenotype. Npj Biofilms Microbiomes. 2020 Jan 10;6(1):2.
17. Nordstedt NP, Chapin LJ, Taylor CG, Jones ML. Identification of *Pseudomonas* Spp. That Increase Ornamental Crop Quality During Abiotic Stress. Front Plant Sci. 2020 Jan 28;10:1754.
18. Pandey S, Gupta S. Evaluation of *Pseudomonas* sp. for its multifarious plant growth promoting potential and its ability to alleviate biotic and abiotic stress in tomato (*Solanum lycopersicum*) plants. Sci Rep. 2020 Dec 1;10(1):20951.
19. Sah S, Krishnani S, Singh R. *Pseudomonas* mediated nutritional and growth promotional activities for sustainable food security. Curr Res Microb Sci. 2021 Dec;2:100084.
20. Zheng Y, Cao X, Zhou Y, Ma S, Wang Y, Li Z, et al. Purines enrich root-associated *Pseudomonas* and improve wild soybean growth under salt stress. Nat Commun. 2024 Apr 25;15(1):3520.
21. Qin S, Xiao W, Zhou C, Pu Q, Deng X, Lan L, et al. *Pseudomonas aeruginosa*: pathogenesis, virulence factors, antibiotic resistance, interaction with host, technology advances and emerging therapeutics. Signal Transduct Target Ther. 2022 Jun 25;7(1):199.
22. Bakki M, Banane B, Marhane O, Esmaeel Q, Hatimi A, Barka EA, et al. Phosphate solubilizing *Pseudomonas* and *Bacillus* combined with rock phosphates promoting tomato growth and reducing bacterial canker disease. Front Microbiol. 2024 May 3;15:1289466.
23. Blanco-Vargas A, Rodríguez-Gacha LM, Sánchez-Castro N, Garzón-Jaramillo R, Pedroza-Camacho LD, Poutou-Piñales RA, et al. Phosphate-solubilizing *Pseudomonas* sp., and

Serratia sp., co-culture for *Allium cepa* L. growth promotion. Heliyon. 2020 Oct;6(10):e05218.

24. Chen J, Zhao G, Wei Y, Dong Y, Hou L, Jiao R. Isolation and screening of multifunctional phosphate solubilizing bacteria and its growth-promoting effect on Chinese fir seedlings. Sci Rep. 2021 Apr 27;11(1):9081.
25. Faller L, Leite MFA, Kuramae EE. Enhancing phosphate-solubilising microbial communities through artificial selection. Nat Commun. 2024 Feb 23;15(1):1649.
26. Ou K, He X, Cai K, Zhao W, Jiang X, Ai W, et al. Phosphate-Solubilizing *Pseudomonas* sp. Strain WS32 Rhizosphere Colonization-Induced Expression Changes in Wheat Roots. Front Microbiol. 2022 Jun 30;13:927889.
27. Paul D, Sinha SN. Isolation and characterization of phosphate solubilizing bacterium *Pseudomonas aeruginosa* KUPSB12 with antibacterial potential from river Ganga, India. Ann Agrar Sci. 2017 Mar;15(1):130–6.
28. Grosse C, Brandt N, Van Antwerpen P, Wintjens R, Matthijs S. Two new siderophores produced by *Pseudomonas* sp. NCIMB 10586: The anti-oomycete non-ribosomal peptide synthetase-dependent mupirochelin and the NRPS-independent triabactin. Front Microbiol. 2023 Mar 24;14:1143861.
29. Jin Z, Li J, Ni L, Zhang R, Xia A, Jin F. Conditional privatization of a public siderophore enables *Pseudomonas aeruginosa* to resist cheater invasion. Nat Commun. 2018 Apr 11;9(1):1383.
30. Luján AM, Gómez P, Buckling A. Siderophore cooperation of the bacterium *Pseudomonas fluorescens* in soil. Biol Lett. 2015 Feb;11(2):20140934.

31. Song Y, Wu X, Li Z, Ma QQ, Bao R. Molecular mechanism of siderophore regulation by the *Pseudomonas aeruginosa* BfmRS two-component system in response to osmotic stress. Commun Biol. 2024 Mar 9;7(1):295.

32. Syed A, Elgorban AM, Bahkali AH, Eswaramoorthy R, Iqbal RK, Danish S. Metal-tolerant and siderophore producing *Pseudomonas* fluorescence and *Trichoderma* spp. improved the growth, biochemical features and yield attributes of chickpea by lowering Cd uptake. Sci Rep. 2023 Mar 18;13(1):4471.

33. Chi SI, Akuma M, Xu R, Plante V, Hadinezhad M, Tambong JT. Phenazines are involved in the antagonism of a novel subspecies of *Pseudomonas chlororaphis* strain S1Bt23 against *Pythium ultimum*. Sci Rep. 2024 Sep 3;14(1):20517.

34. Garbeva P, Silby MW, Raaijmakers JM, Levy SB, De Boer W. Transcriptional and antagonistic responses of *Pseudomonas fluorescens* Pf0-1 to phylogenetically different bacterial competitors. ISME J. 2011 Jun 1;5(6):973–85.

35. Mehmood N, Saeed M, Zafarullah S, Hyder S, Rizvi ZF, Gondal AS, et al. Multifaceted Impacts of Plant-Beneficial *Pseudomonas* spp. in Managing Various Plant Diseases and Crop Yield Improvement. ACS Omega. 2023 Jun 27;8(25):22296–315.

36. Neve RL, Giedraitis E, Akbari MS, Cohen S, Phelan VV. Secondary metabolite profiling of *Pseudomonas aeruginosa* isolates reveals rare genomic traits. Porto C, editor. mSystems. 2024 May 16;9(5):e00339-24.

37. Gomila M, PeÃ±a A, Mulet M, Lalucat J, GarcÃ-a-ValdÃ©s E. Phylogenomics and systematics in *Pseudomonas*. Front Microbiol [Internet]. 2015 Mar 18 [cited 2025 Jan 22];6. Available from:

http://www.frontiersin.org/Evolutionary_and_Genomic_Microbiology/10.3389/fmicb.2015.00214/abstract

38. Gomila M, Busquets A, Mulet M, García-Valdés E, Lalucat J. Clarification of Taxonomic Status within the *Pseudomonas syringae* Species Group Based on a Phylogenomic Analysis. Front Microbiol. 2017 Dec 7;8:2422.

39. Hu S, Li X, Yin X, Li R, Zhang R, Zang J, et al. Species-specific identification of *Pseudomonas* based on 16S–23S rRNA gene internal transcribed spacer (ITS) and its combined application with next-generation sequencing. BMC Microbiol. 2022 Dec;22(1):188.

40. Scales BS, Dickson RP, LiPuma JJ, Huffnagle GB. Microbiology, Genomics, and Clinical Significance of the *Pseudomonas fluorescens* Species Complex, an Unappreciated Colonizer of Humans. Clin Microbiol Rev. 2014 Oct;27(4):927–48.

41. Ferrer Obiol J, Herranz JM, Paris JR, Whiting JR, Rozas J, Riutort M, et al. Species delimitation using genomic data to resolve taxonomic uncertainties in a speciation continuum of pelagic seabirds. Mol Phylogenet Evol. 2023 Feb;179:107671.

42. Lalucat J, Mulet M, Gomila M, García-Valdés E. Genomics in Bacterial Taxonomy: Impact on the Genus *Pseudomonas*. Genes. 2020 Jan 29;11(2):139.

43. Puigbò P, Wolf YI, Koonin EV. Genome-Wide Comparative Analysis of Phylogenetic Trees: The Prokaryotic Forest of Life. In: Anisimova M, editor. Evolutionary Genomics [Internet]. Totowa, NJ: Humana Press; 2012 [cited 2025 Jan 22]. p. 53–79. (Methods in Molecular Biology; vol. 856). Available from: https://link.springer.com/10.1007/978-1-61779-585-5_3

44. Jain C, Rodriguez-R LM, Phillippy AM, Konstantinidis KT, Aluru S. High throughput ANI analysis of 90K prokaryotic genomes reveals clear species boundaries. Nat Commun. 2018 Nov 30;9(1):5114.
45. Richter M, Rosselló-Móra R. Shifting the genomic gold standard for the prokaryotic species definition. Proc Natl Acad Sci. 2009 Nov 10;106(45):19126–31.
46. Rodriguez-R LM, Conrad RE, Viver T, Feistel DJ, Lindner BG, Venter SN, et al. An ANI gap within bacterial species that advances the definitions of intra-species units. Jouline IB, editor. mBio. 2024 Jan 16;15(1):e02696-23.
47. Thompson CC, Chimetto L, Edwards RA, Swings J, Stackebrandt E, Thompson FL. Microbial genomic taxonomy. BMC Genomics. 2013; 14: 913. doi:10.1186/1471-2164-14-913.
48. Auch AF, von Jan M, Klenk HP, Göker M. Digital DNA-DNA hybridization for microbial species delineation by means of genome-to-genome sequence comparison. Standards in Genomic Sciences. 2010; 2(1):117–34. doi:10.4056/sigs.531120
49. Meier-Kolthoff JP, Auch AF, Klenk HP, Göker M. Genome sequence-based species delimitation with confidence intervals and improved distance functions. BMC Bioinformatics. 2013 Dec;14(1):60.
50. Auch AF, Klenk HP, Göker M. Standard operating procedure for calculating genome-to-genome distances based on high-scoring segment pairs. Stand Genomic Sci. 2010 Jan 28;2(1):142–8.
51. Church DL, Cerutti L, Gürtler A, Griener T, Zelazny A, Emler S. Performance and Application of 16S rRNA Gene Cycle Sequencing for Routine Identification of Bacteria in the Clinical Microbiology Laboratory. Clin Microbiol Rev. 2020 Sep 16;33(4):e00053-19.

52. Clarridge JE. Impact of 16S rRNA Gene Sequence Analysis for Identification of Bacteria on Clinical Microbiology and Infectious Diseases. Clin Microbiol Rev. 2004 Oct;17(4):840–62.

53. Hassler HB, Probert B, Moore C, Lawson E, Jackson RW, Russell BT, et al. Phylogenies of the 16S rRNA gene and its hypervariable regions lack concordance with core genome phylogenies. Microbiome. 2022 Dec;10(1):104.

54. Gabriel MW, Matsui GY, Friedman R, Lovell CR. Optimization of Multilocus Sequence Analysis for Identification of Species in the Genus *Vibrio*. Elkins CA, editor. Appl Environ Microbiol. 2014 Sep;80(17):5359–65.

55. Korczak B, Christensen H, Emler S, Frey J, Kuhnert P. Phylogeny of the family Pasteurellaceae based on *rpoB* sequences. Int J Syst Evol Microbiol. 2004 Jul 1;54(4):1393–9.

56. López-Hermoso C, De La Haba RR, Sánchez-Porro C, Papke RT, Ventosa A. Assessment of MultiLocus Sequence Analysis as a Valuable Tool for the Classification of the Genus *Salinivibrio*. Front Microbiol. 2017 Jun 22;8:1107.

57. Mulet M, Bennasar A, Lalucat J, García-Valdés E. An *rpoD*-based PCR procedure for the identification of *Pseudomonas* species and for their detection in environmental samples. Mol Cell Probes. 2009 Jun;23(3–4):140–7.

58. Yáñez MA, Catalán V, Apráiz D, Figueras MJ, Martínez-Murcia AJ. Phylogenetic analysis of members of the genus *Aeromonas* based on *gyrB* gene sequences. Int J Syst Evol Microbiol. 2003 May 1;53(3):875–83.

59. Moule AL, Wilkinson SG. Polar Lipids, Fatty Acids, and Isoprenoid Quinones of *Alteromonas putrefaciens* (*Shewanella putrefaciens*). Syst Appl Microbiol. 1987 Aug;9(3):192–8.

60. Yamano R, Yu J, Jiang C, Harjuno Condro Haditomo A, Mino S, Sakai Y, et al. Taxonomic revision of the genus *Amphritea* supported by genomic and in silico chemotaxonomic analyses, and the proposal of *Aliamphritea* gen. nov. Yurchenko V, editor. PLOS ONE. 2022 Aug 10;17(8):e0271174.

61. Zou Y, Lin X, Xue W, Tuo L, Chen MS, Chen XH, et al. Characterization and description of *Faecalibacterium butyricigenerans* sp. nov. and *F. longum* sp. nov., isolated from human faeces. Sci Rep. 2021 May 31;11(1):11340.

62. Lacey RF, Sullivan-Hill BA, Deslippe JR, Keyzers RA, Gerth ML. The Fatty Acid Methyl Ester (FAME) profile of *Phytophthora agathidicida* and its potential use as diagnostic tool. FEMS Microbiol Lett. 2021 Sep 8;368(17):fnab113.

63. Heyrman J, Mergaert J, Denys R, Swings J. The use of fatty acid methyl ester analysis (FAME) for the identification of heterotrophic bacteria present on three mural paintings showing severe damage by microorganisms. FEMS Microbiol Lett. 1999 Dec;181(1):55–62.

64. Inglis TJJ, Aravena-Roman M, Ching S, Croft K, Wuthiekanun V, Mee BJ. Cellular Fatty Acid Profile Distinguishes *Burkholderia pseudomallei* from Avirulent *Burkholderia thailandensis*. J Clin Microbiol. 2003 Oct;41(10):4812–4.

65. Rees CA, Beccaria M, Franchina FA, Hill JE, Purcaro G. Fatty Acid Methyl Ester (FAME) Profiling Identifies Carbapenemase-Producing *Klebsiella pneumoniae* Belonging to Clonal Complex 258. Separations. 2019 Jun 17;6(2):32.

66. Vauterin L, Yang P, Swings J. Utilization of Fatty Acid Methyl Esters for the Differentiation of New *Xanthomonas* Species. Int J Syst Bacteriol. 1996 Jan 1;46(1):298–304.

67. Kunitsky C, Osterhout G, Sasser M. Identification of microorganisms using fatty acid methyl ester (FAME) analysis and the MIDI Sherlock® microbial identification system. Newark (DE): MIDI, Inc.; 2006.

68. Kim M, Song J, Shin SY, Kogure K, Kang I, Cho JC. Cultivation of deep-sea bacteria from the Northwest Pacific Ocean and characterization of *Limnobacter profundi* sp. nov., a phenol-degrading bacterium. Front Mar Sci. 2024 Nov 4;11:1449548.

69. Tareen S, Risdian C, Müsken M, Wink J. *Qipengyuania pacifica* sp. nov., a Novel Carotenoid-Producing Marine Bacterium of the Family Erythrobacteraceae, Isolated from Sponge (Demospongiae), and Antimicrobial Potential of Its Crude Extract. Diversity. 2022 Apr 14;14(4):295.

70. Yin LZ, Li JL, Fang BZ, Liu ZT, Wang P, Dong L, et al. *Roseomonas ponticola* sp. nov., a novel bacterium isolated from Pearl River estuary. Int J Syst Evol Microbiol [Internet]. 2021 Oct 20 [cited 2025 Jan 22];71(10).

71. Yin LZ, Li JL, Fang BZ, Liu ZT, Wang P, Dong L, et al. *Roseomonas ponticola* sp. nov., a novel bacterium isolated from Pearl River estuary. Int J Syst Evol Microbiol [Internet]. 2021 Oct 20 [cited 2025 Jan 22];71(10).

72. Matz C, Deines P, JÃ¼rgens K. Phenotypic variation in *Pseudomonas* sp. CM10 determines microcolony formation and survival under protozoan grazing. FEMS Microbiol Ecol. 2002 Jan;39(1):57–65.

73. Orellana-Saez M, Pacheco N, Costa JI, Mendez KN, Miossec MJ, Meneses C, et al. In-Depth Genomic and Phenotypic Characterization of the Antarctic Psychrotolerant Strain *Pseudomonas* sp. MPC6 Reveals Unique Metabolic Features, Plasticity, and Biotechnological Potential. Front Microbiol. 2019 May 24;10:1154.

74. Jia J, Wang X, Deng P, Ma L, Baird SM, Li X, et al. *Pseudomonas glycinae* sp. nov. isolated from the soybean rhizosphere. MicrobiologyOpen. 2020 Sep;9(9):e1101.

75. Bouteiller M, Dupont C, Bourigault Y, Latour X, Barbey C, Konto-Ghiorghi Y, et al. *Pseudomonas Flagella*: Generalities and Specificities. Int J Mol Sci. 2021 Mar 24;22(7):3337.

76. Kearns DB. A field guide to bacterial swarming motility. Nat Rev Microbiol. 2010 Sep;8(9):634–44.

77. Kühn MJ, Schmidt FK, Farthing NE, Rossmann FM, Helm B, Wilson LG, et al. Spatial arrangement of several flagellins within bacterial flagella improves motility in different environments. Nat Commun. 2018 Dec 18;9(1):5369.

78. Bartholomew JW, Mittwer T. The Gram Stain. Bacteriol Rev. 1952 Mar;16(1):1–29.

79. Mrazova K, Bacovsky J, Sedrlova Z, Slaninova E, Obruca S, Fritz I, et al. Urany-Less Low Voltage Transmission Electron Microscopy: A Powerful Tool for Ultrastructural Studying of Cyanobacterial Cells. Microorganisms. 2023 Mar 29;11(4):888.

80. Crespi R, Barone A, Covani U, Ciaglia RN, Romanos GE. Effects of CO_2 Laser Treatment on Fibroblast Attachment to Root Surfaces. A Scanning Electron Microscopy Analysis. J Periodontol. 2002 Nov;73(11):1308–12.

81. Siqueira JF, Lopes HP. Bacteria on the apical root surfaces of untreated teeth with periradicular lesions: a scanning electron microscopy study. Int Endod J. 2001 Apr;34(3):216–20.
82. Shotton DM. Review: Video-enhanced light microscopy and its applications in cell biology. J Cell Sci. 1988 Feb 1;89(2):129–50.
83. Lamichhane JR, Varvaro L. A new medium for the detection of fluorescent pigment production by pseudomonads. Plant Pathol. 2013 Jun;62(3):624–32.
84. Mira P, Yeh P, Hall BG. Estimating microbial population data from optical density. Omri A, editor. PLOS ONE. 2022 Oct 13;17(10):e0276040.
85. Sezonov G, Joseleau-Petit D, D'Ari R. *Escherichia coli* Physiology in Luria-Bertani Broth. J Bacteriol. 2007 Dec;189(23):8746–9.
86. Zhang K, Ding W, Han C, Long L, Yin H, Yin J. Investigation on taxonomy, secondary metabolites and antibacterial activity of *Streptomyces sediminicola* sp. nov., a novel marine sediment-derived Actinobacteria. Microb Cell Factories. 2024 Oct 19;23(1):285.
87. Ahmad Z, Wu J, Chen L, Dong W. Isolated Bacillus subtilis strain 330-2 and its antagonistic genes identified by the removing PCR. Sci Rep. 2017 May 11;7(1):1777.
88. Ngalimat MS, Raja Abd. Rahman RNZ, Yusof MT, Syahir A, Sabri S. Characterisation of bacteria isolated from the stingless bee, *Heterotrigona itama*, honey, bee bread and propolis. PeerJ. 2019 Aug 22;7:e7478.
89. Woźniak M, Tyśkiewicz R, Siebielec S, Gałązka A, Jaroszuk-Ściseł J. Metabolic Profiling of Endophytic Bacteria in Relation to Their Potential Application as Components of Multi-Task Biopreparations. Microb Ecol. 2023 Nov;86(4):2527–40.

90. Wu P, Yuan Q, Cheng T, Han Y, Zhao W, Liao X, et al. Genome sequencing and metabolic network reconstruction of a novel sulfur-oxidizing bacterium *Acidithiobacillus ameehan*. Front Microbiol. 2023 Nov 20;14:1277847.

91. Chelius MK, Triplett EW. Immunolocalization of Dinitrogenase Reductase Produced by *Klebsiella pneumoniae* in Association with *Zea mays* L. Appl Environ Microbiol. 2000 Feb;66(2):783–7.

92. Shin HB, Yoon J, Lee Y, Kim MS, Lee K. Comparison of MALDI-TOF MS, Housekeeping Gene Sequencing, and 16S rRNA Gene Sequencing for Identification of *Aeromonas* Clinical Isolates. Yonsei Med J. 2015;56(2):550.

93. Gouy M, Guindon S, Gascuel O. SeaView Version 4: A Multiplatform Graphical User Interface for Sequence Alignment and Phylogenetic Tree Building. Mol Biol Evol. 2010 Feb 1;27(2):221–4.

94. Katoh K, editor. Multiple Sequence Alignment: Methods and Protocols [Internet]. New York, NY: Springer US; 2021 [cited 2025 Jan 22]. (Methods in Molecular Biology; vol. 2231). Available from: https://link.springer.com/10.1007/978-1-0716-1036-7

95. Felsenstein J. Evolutionary trees from DNA sequences: A maximum likelihood approach. J Mol Evol. 1981 Nov;17(6):368–76.

96. Guindon S, Gascuel O. A Simple, Fast, and Accurate Algorithm to Estimate Large Phylogenies by Maximum Likelihood. Rannala B, editor. Syst Biol. 2003 Oct 1;52(5):696–704.

97. Jacobs J, Nakamoto A, Mastoras M, Loucks H, Mirchandani C, Karim L, et al. Complete de novo assembly of Wolbachia endosymbiont of *Drosophila willistoni* using long-read genome sequencing. Sci Rep. 2024 Aug 1;14(1):17770.

98. Krumsiek J, Arnold R, Rattei T. Gepard: a rapid and sensitive tool for creating dotplots on genome scale. Bioinformatics. 2007 Apr 15;23(8):1026–8.

99. Grant JR, Stothard P. The CGView Server: a comparative genomics tool for circular genomes. Nucleic Acids Res. 2008 May 19;36(Web Server):W181–4.

100. Stothard P, Grant JR, Van Domselaar G. Visualizing and comparing circular genomes using the CGView family of tools. Brief Bioinform. 2019 Jul 19;20(4):1576–82.

101. Tatusova T, DiCuccio M, Badretdin A, Chetvernin V, Nawrocki EP, Zaslavsky L, et al. NCBI prokaryotic genome annotation pipeline. Nucleic Acids Res. 2016 Aug 19;44(14):6614–24.

102. Meier-Kolthoff JP, Carbasse JS, Peinado-Olarte RL, Göker M. TYGS and LPSN: a database tandem for fast and reliable genome-based classification and nomenclature of prokaryotes. Nucleic Acids Res. 2022 Jan 7;50(D1):D801–7.

103. Meier-Kolthoff JP, Göker M. TYGS is an automated high-throughput platform for state-of-the-art genome-based taxonomy. Nat Commun. 2019 May 16;10(1):2182.

104. Ondov BD, Treangen TJ, Melsted P, Mallonee AB, Bergman NH, Koren S, et al. Mash: fast genome and metagenome distance estimation using MinHash. Genome Biol. 2016 Dec;17(1):132.

105. Lagesen K, Hallin P, Rødland EA, Stærfeldt HH, Rognes T, Ussery DW. RNAmmer: consistent and rapid annotation of ribosomal RNA genes. Nucleic Acids Res. 2007 May;35(9):3100–8.

106. Camacho C, Coulouris G, Avagyan V, Ma N, Papadopoulos J, Bealer K, et al. BLAST+: architecture and applications. BMC Bioinformatics. 2009 Dec;10(1):421.

107. Lefort V, Desper R, Gascuel O. FastME 2.0: A Comprehensive, Accurate, and Fast Distance-Based Phylogeny Inference Program: Table 1. Mol Biol Evol. 2015 Oct;32(10):2798–800.

108. Farris JS. Estimating Phylogenetic Trees from Distance Matrices. Am Nat. 1972 Sep;106(951):645–68.

109. Kreft Ł, Botzki A, Coppens F, Vandepoele K, Van Bel M. PhyD3: a phylogenetic tree viewer with extended phyloXML support for functional genomics data visualization. Kelso J, editor. Bioinformatics. 2017 Sep 15;33(18):2946–7.

110. Meier-Kolthoff JP, Hahnke RL, Petersen J, Scheuner C, Michael V, Fiebig A, et al. Complete genome sequence of DSM 30083T, the type strain (U5/41T) of *Escherichia coli*, and a proposal for delineating subspecies in microbial taxonomy. Stand Genomic Sci. 2014 Dec 8;9(1):2.

111. Chang DH, Rhee MS, Kim JS, Lee Y, Park MY, Kim H, et al. *Pseudomonas kribbensis* sp. nov., isolated from garden soils in Daejeon, Korea. Antonie Van Leeuwenhoek. 2016 Nov;109(11):1433–46.

112. Pascual J, García-López M, Bills GF, Genilloud O. *Pseudomonas granadensis* sp. nov., a new bacterial species isolated from the Tejeda, Almijara and Alhama Natural Park, Granada, Spain. Int J Syst Evol Microbiol. 2015 Feb 1;65(Pt_2):625–32.

113. Tvrzová L, Schumann P, Spröer C, Sedláček I, Páčová Z, Šedo O, et al. *Pseudomonas moraviensis* sp. nov. and *Pseudomonas vranovensis* sp. nov., soil bacteria isolated on nitroaromatic compounds, and emended description of *Pseudomonas asplenii*. Int J Syst Evol Microbiol. 2006 Nov 1;56(11):2657–63.

114. López JR, Diéguez AL, Doce A, De La Roca E, De La Herran R, Navas JI, et al. *Pseudomonas baetica* sp. nov., a fish pathogen isolated from wedge sole, *Dicologlossa cuneata* (Moreau). Int J Syst Evol Microbiol. 2012 Apr 1;62(Pt_4):874–82.
115. Cámara B, Strömpl C, Verbarg S, Spröer C, Pieper DH, Tindall BJ. *Pseudomonas reinekei* sp. nov., *Pseudomonas moorei* sp. nov. and *Pseudomonas mohnii* sp. nov., novel species capable of degrading chlorosalicylates or isopimaric acid. Int J Syst Evol Microbiol. 2007 May 1;57(5):923–31.
116. Caudill MT, Brayton KA. The Use and Limitations of the 16S rRNA Sequence for Species Classification of Anaplasma Samples. Microorganisms. 2022 Mar 12;10(3):605.
117. Poblete-Morales M, Carvajal D, Almasia R, Michea S, Cantillana C, Levican A, et al. *Pseudomonas atacamensis* sp. nov., isolated from the rhizosphere of desert bloom plant in the region of Atacama, Chile. Antonie Van Leeuwenhoek. 2020 Aug;113(8):1201–11.
118. Hrovat K, Dutilh BE, Medema MH, Melkonian C. Taxonomic resolution of different 16S rRNA variable regions varies strongly across plant-associated bacteria. ISME Commun. 2024 Jan 8;4(1):ycae034.
119. Bartoš O, Chmel M, Swierczková I. The overlooked evolutionary dynamics of 16S rRNA revises its role as the “gold standard” for bacterial species identification. Sci Rep. 2024 Apr 20;14(1):9067.
120. Salvà-Serra F, Jakobsson HE, Busquets A, Gomila M, Jaén-Luchoro D, Seguí C, et al. Genome Sequences of Two Naphthalene-Degrading Strains of *Pseudomonas balearica*, Isolated from Polluted Marine Sediment and from an Oil Refinery Site. Genome Announc. 2017 Apr 6;5(14):e00116-17.

121. Wiehlmann L, Wagner G, Cramer N, Siebert B, Gudowius P, Morales G, et al. Population structure of *Pseudomonas aeruginosa*. Proc Natl Acad Sci. 2007 May 8;104(19):8101–6.

122. Atanasov KE, Galbis DM, Cornadó D, Serpico A, Sánchez G, Bosch M, et al. *Pseudomonas fitomaticsae* sp. nov., isolated at Marimurtra Botanical Garden in Blanes, Catalonia, Spain. Int J Syst Evol Microbiol. 2022 Oct 19;72(10).

123. Girard L, Lood C, Höfte M, Vandamme P, Rokni-Zadeh H, Van Noort V, et al. The Ever-Expanding *Pseudomonas* Genus: Description of 43 New Species and Partition of the *Pseudomonas putida* Group. Microorganisms. 2021 Aug 18;9(8):1766.

124. Hesse C, Schulz F, Bull CT, Shaffer BT, Yan Q, Shapiro N, et al. Genome-based evolutionary history of *Pseudomonas* spp. Environ Microbiol. 2018 Jun;20(6):2142–59.

125. Testerman T, Varga J, Schiffer MM, Donohue H, Vieira Da Silva C, Graf J. *Pseudomonas aphyarum* sp. nov., *Pseudomonas fontis* sp. nov., *Pseudomonas idahonensis* sp. nov. and *Pseudomonas rubra* sp. nov., isolated from in, and around, a rainbow trout farm. Int J Syst Evol Microbiol. 2023 Dec 18;73(12).

126. Todorović I, Abrouk D, Kyselková M, Lavire C, Rey M, Raičević V, et al. Two novel species isolated from wheat rhizospheres in Serbia: *Pseudomonas serbica* sp. nov. and *Pseudomonas serboccidentalis* sp. nov. Syst Appl Microbiol. 2023 Jul;46(4):126425.

127. Ling MH. Core Pseudomonas genome from 10 *pseudomonas* species. MOJ Proteomics Bioinforma. 2020 Jul 17;9(3).

128. Jin Y, Zhou J, Zhou J, Hu M, Zhang Q, Kong N, et al. Genome-based classification of *Burkholderia cepacia* complex provides new insight into its taxonomic status. Biol Direct. 2020 Dec;15(1):6.

129. Ciufo S, Kannan S, Sharma S, Badretdin A, Clark K, Turner S, et al. Using average nucleotide identity to improve taxonomic assignments in prokaryotic genomes at the NCBI. Int J Syst Evol Microbiol. 2018 Jul 1;68(7):2386–92.

130. Versmessen N, Mispelaere M, Vandekerckhove M, Hermans C, Boelens J, Vranckx K, et al. Average Nucleotide Identity and Digital DNA-DNA Hybridization Analysis Following PromethION Nanopore-Based Whole Genome Sequencing Allows for Accurate Prokaryotic Typing. Diagnostics. 2024 Aug 17;14(16):1800.

131. Kwon SW, Kim JS, Park IC, Yoon SH, Park DH, Lim CK, et al. *Pseudomonas koreensis* sp. nov., *Pseudomonas umsongensis* sp. nov. and *Pseudomonas jinjuensis* sp. nov., novel species from farm soils in Korea. Int J Syst Evol Microbiol. 2003 Jan 1;53(1):21–7.

132. Morimoto Y, Lu YJ, Zuo H, Aibibula Z, Tohya M, Kirikae T, et al. *Pseudomonas allokribbensis* sp. nov. and *Pseudomonas gozinkensis* sp. nov., Two New Species Isolated from a Volcanic Island, Izu Oshima, Japan. Curr Microbiol. 2021 Apr;78(4):1670–7.

P1. Ali MS, Mony FTZ, Eisenback JD. Surface Sterilization of Soybean Nodules for Microbiological Studies. *protocols.io*, 2025a. https://www.protocols.io/private/3F5FACE8049811F0B4580A58A9FEAC02

P2. Ali MS, Mony FTZ, Eisenback JD. Selective King's B Medium for Gram Negative Bacteria. *protocols.io*, 2025b. https://www.protocols.io/private/9FFDB692048E11F0A6C00A58A9FEAC02

P3. Ali MS, Mony FTZ, Eisenback JD. LB lite Growth Media Protocol. *protocols.io*, 2025c. https://www.protocols.io/private/E2F913ED044011F0A7330A58A9FEAC02

P4. Ali MS, Mony FTZ, Eisenback JD. Glycerol Stock Solution Preparation for Long-Term Bacterial Storage. *protocols.io*, 2025d. https://www.protocols.io/private/5C456094049E11F0A6C00A58A9FEAC02

P5. Ali MS, Mony FTZ, Eisenback JD. Transmission Electron Microscopy (TEM) Protocol for *Pseudomonas* sp. *protocols.io*, 2025f. https://www.protocols.io/private/A9FB11B9055B11F0B0AB0A58A9FEAC02

P6. Ali MS, Mony FTZ, Eisenback JD. Identification of *Pseudomonas* sp. using fatty acid methyl ester (FAME) analysis. *protocols.io*, 2025g. https://www.protocols.io/private/714A65A7056511F0B0AB0A58A9FEAC02

P7. Ali MS, Mony FTZ, Eisenback JD. Identification of Bacteria using BIOLOG GEN III Assay. *protocols.io*, 2025h. https://www.protocols.io/private/B4B9CBFA059511F082780A58A9FEAC02

P8. Ali MS, Mony FTZ, Eisenback JD. Genomic DNA Extraction Protocol using DNeasy® UltraClean® Microbial Kit. *protocols.io*, 2025i. https://www.protocols.io/private/4D8344B004F311F0B0AB0A58A9FEAC02

P9. Ali MS, Mony FTZ, Eisenback JD. Agarose Gel Electrophoresis for DNA Analysis. *protocols.io*, 2025j. https://www.protocols.io/private/801EE22204E211F0A6C00A58A9FEAC02

P10. Ali MS, Mony FTZ, Eisenback JD. PCR Run Protocol for Bacterial 16S rRNA Gene Amplification. *protocols.io*, 2025k. https://www.protocols.io/private/C8D78DC0055411F0B0AB0A58A9FEAC02

Supporting information

S1 Fig. Comparative fluorescence of JDE115 and *Klebsiella* sp. This image highlights the fluorescence capability of ‘*Candidatus* Pseudomonas auctus’ nov. sp. JDE115, evident in three fluorescent plates, compared to the non-fluorescent Klebsiella sp. The test confirms the fluorescence property of JDE115 under UV light.

S1 Movie. Live observation of ‘*Candidatus* Pseudomonas auctus’ nov. sp. JDE115 demonstrating active motility. This movie was recorded at 630x using phase contrast, causing the bacteria to appear black; the lighting was gradually changed to bright field to make the bacteria appear white and the clear polysaccharide capsule to become visible.

S2 Fig. Morphological characterization of ‘*Candidatus* Pseudomonas auctus’ JDE115 colonies. The diameters of the colonies were measured with a digital caliper after incubation at 28°C on Luria-Bertani agar for 24hr.

S1 Table. The physiological, morphological, and phenotypic characteristics of ‘*Candidatus* Pseudomonas auctus’ sp. nov. strain JDE115 in Biology GEN III tests. The tests were repeated three times. This analysis also demonstrated the ability of JDE115 to thrive under aerobic conditions that was evident from the tetrazolium dye reduction assay which functions as an indicator of active respiration. In the BIOLOG system, a positive reaction occurs when the bacterium can metabolize a given substrate, leading to dye reduction and a color change from colorless to purple. The widespread positive metabolic activity across multiple carbon sources confirms the presence of an active electron transport chain (ETC), a hallmark of aerobic respiration. Furthermore, the test results indicated the ability of JDE115 to utilize a broad spectrum of organic acids (e.g., L-malic acid, D-malic acid, citric acid, propionic acid, and acetic acid), which are known to be key carbon sources for aerobic bacteria and facultative anaerobes. This metabolic flexibility indicated a bacterium that efficiently engages in oxidative

phosphorylation while still having the capacity to switch to fermentation or alternative electron acceptors when oxygen is limited. The survival of JDE115 under varying NaCl and pH conditions also supports its classification as a facultative aerobe. Its tolerance to 1%, 4%, and 8% NaCl suggests that it can withstand osmotic fluctuations, an important trait for bacteria adapting to different environmental conditions, including those found inside plant tissues.

Figure 1

Click here to access/download;Figure;Fig1.tif

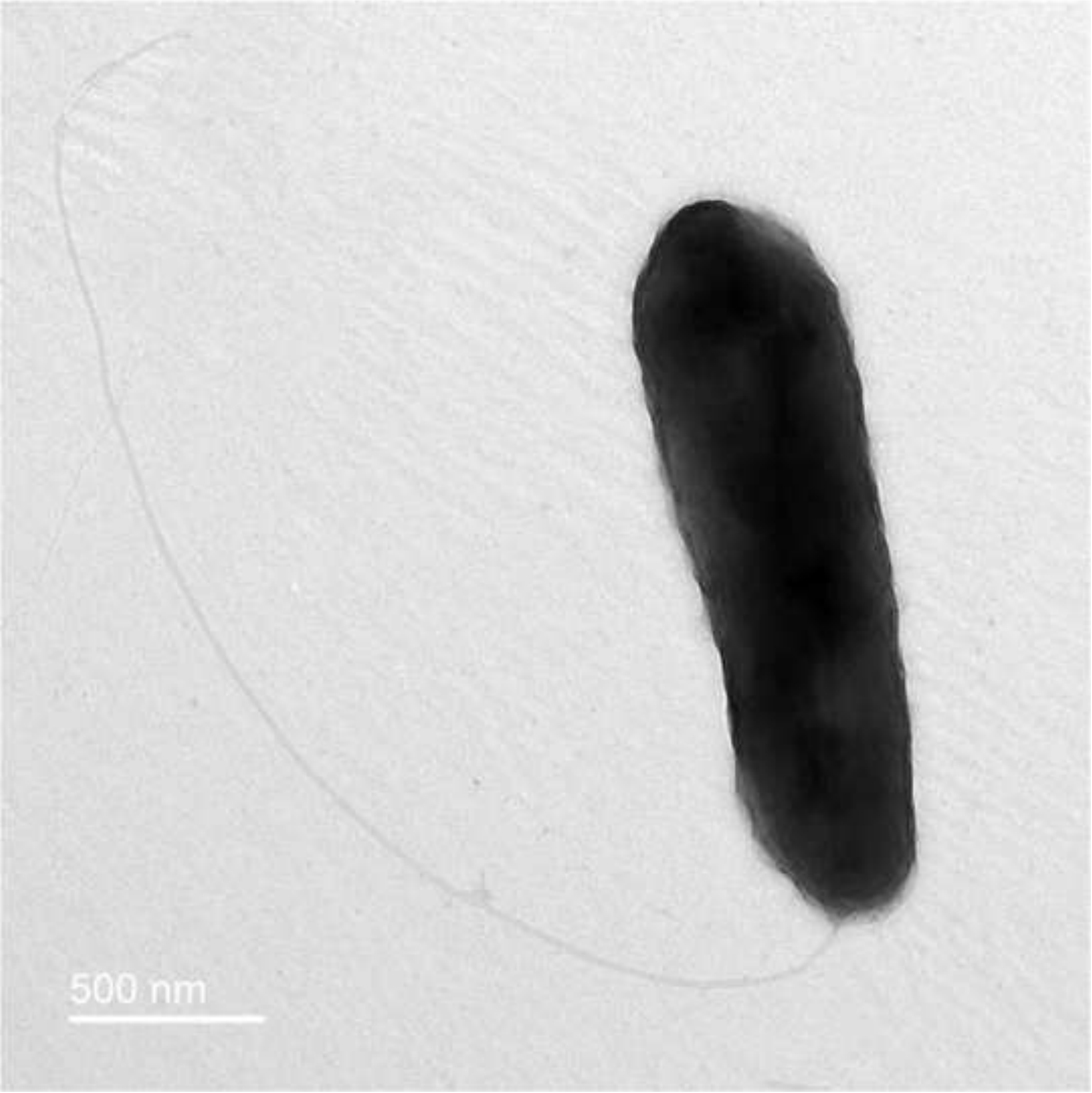

Figure 3

Click here to access/download;Figure;Fig3.tif

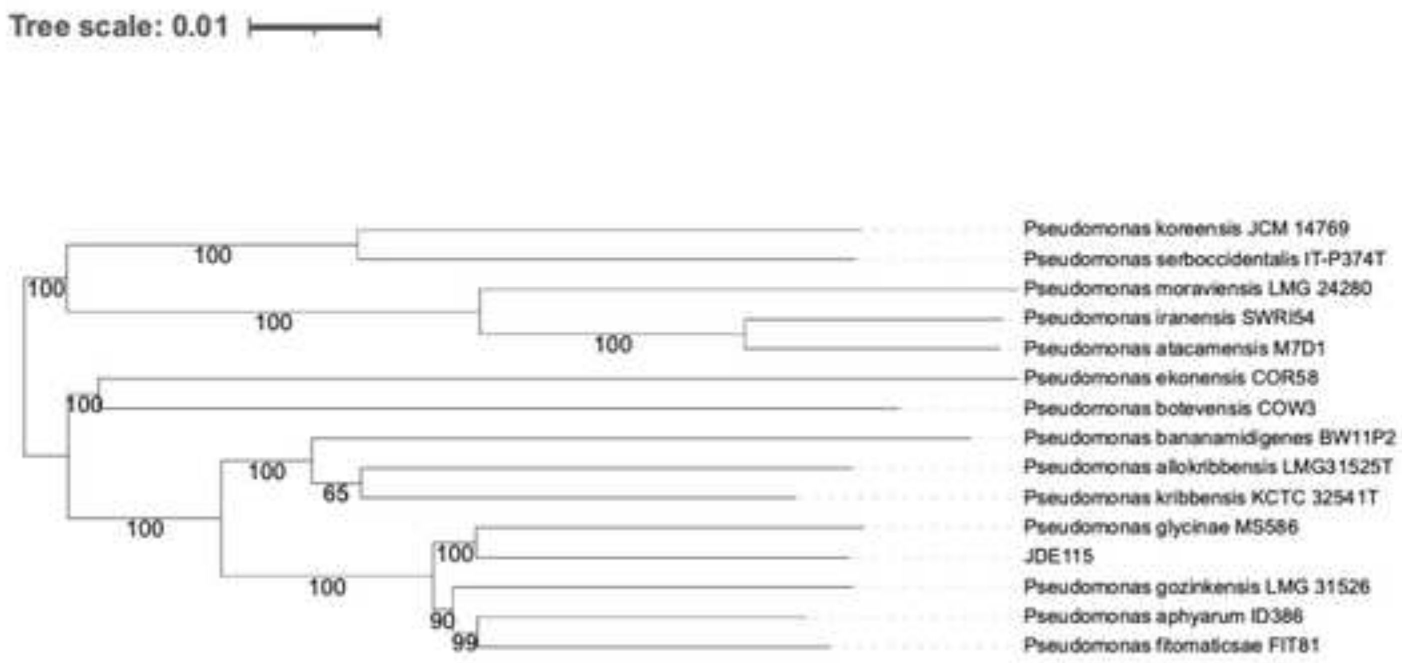

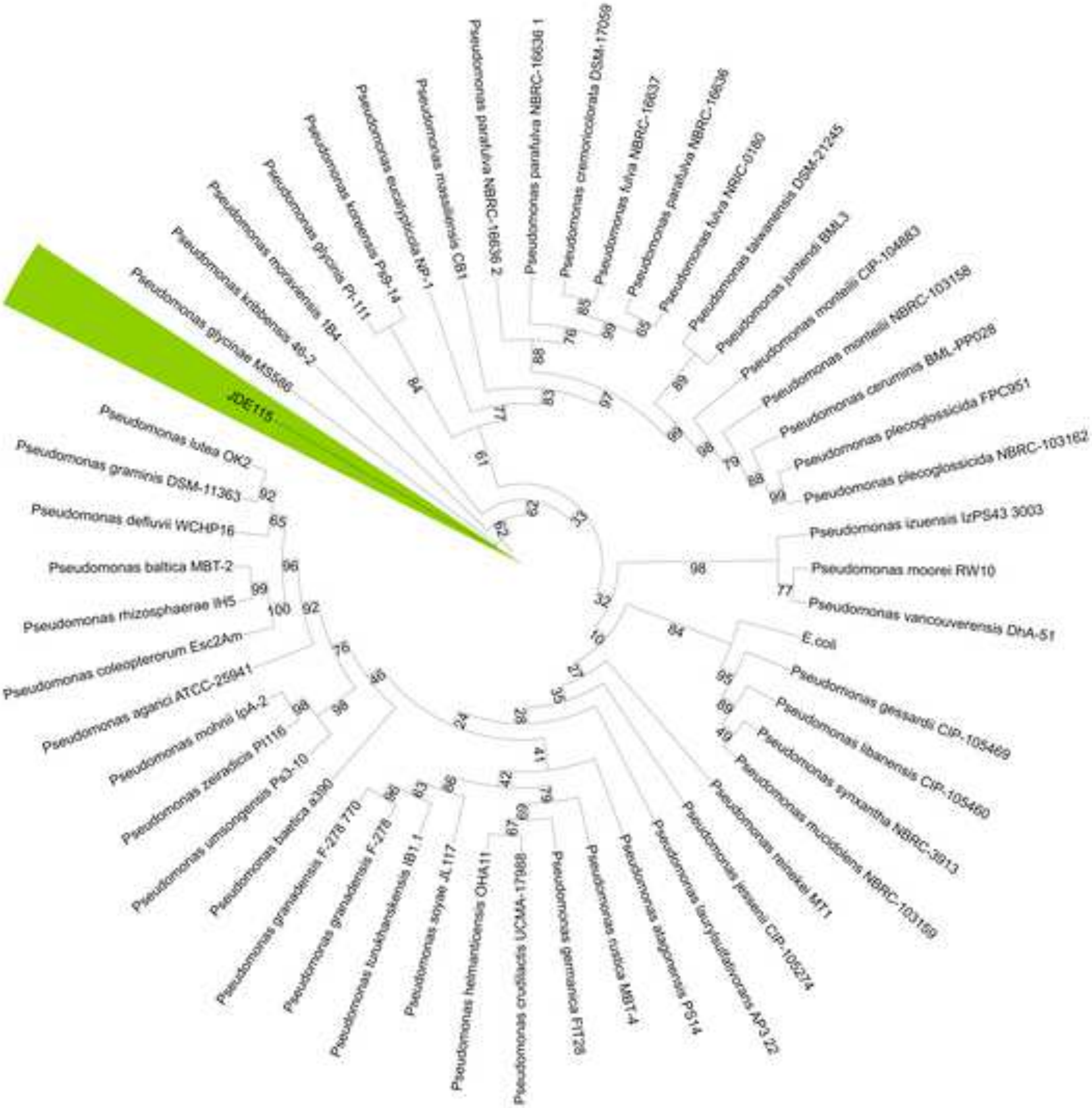
JDE115
Pseudomonas glycinae MS586
Pseudomonas kribbensis 46-2
Pseudomonas moraviensis 1B4
Pseudomonas glycinis Pl-111
Pseudomonas koreensis Ps9-14
Pseudomonas eucalypticola NP-1
Pseudomonas massiliensis CB1
Pseudomonas parafulva NBRC-16636 2
Pseudomonas parafulva NBRC-16636 1
Pseudomonas cremoricolorata DSM-17059
Pseudomonas fulva NBRC-16637
Pseudomonas parafulva NBRC-16636
Pseudomonas fulva NRC-0180
Pseudomonas taiwanensis DSM-21245
Pseudomonas juntendi BML3
Pseudomonas monteilii CIP-104883
Pseudomonas monteilii NBRC-103158
Pseudomonas cerumins BML-PP028
Pseudomonas plecoglossicida FPC951
Pseudomonas plecoglossicida NBRC-103162
Pseudomonas izuensis IzPS43 3003
Pseudomonas moorei RW10
Pseudomonas vancouverensis DhA-51
E.coli
Pseudomonas gessardii CIP-105469
Pseudomonas libanensis CIP-105460
Pseudomonas synxantha NBRC-3913
Pseudomonas mucidolens NBRC-103159
Pseudomonas reinekei MT1
Pseudomonas jessenii CIP-105274
Pseudomonas laurylsulfativorans AP3 22
Pseudomonas atagonensis PS14
Pseudomonas rustica MBT-4
Pseudomonas germanica FIT28
Pseudomonas crudilactis UCMA-17988
Pseudomonas helmanticensis OHA11
Pseudomonas soyae JL117
Pseudomonas turukhanskensis IB1.1
Pseudomonas granadensis F-278
Pseudomonas granadensis F-278 770
Pseudomonas baetica a390
Pseudomonas umsongensis Ps3-10
Pseudomonas zeiradicis PI116
Pseudomonas mohnii IpA-2
Pseudomonas agarici ATCC-25941
Pseudomonas coleopterorum Esc2Am
Pseudomonas rhizosphaerae IH5
Pseudomonas baltica MBT-2
Pseudomonas defluvii WCHP16
Pseudomonas graminis DSM-11363
Pseudomonas lutea OK2

Click here to access/download
Supporting Information
Protocols Published 2025.docx

Supplemental Figure 1

Click here to access/download
Supporting Information
S1 Fig.tif

Supplemental Table 1

Click here to access/download
Supporting Information
S1 Table.docx

Supplemental Figure 2

Click here to access/download
Supporting Information
S2 Fig.tif

Supplemental Movie 1

Click here to access/download

Supporting Information

S1 Movie P. auctus.mp4
