# Supplementary material for: ‘Candidatus Pseudomonas auctus’ sp. nov. JDE115 isolated from nodules on soybean (Glycines max)
Source: PLoS One. 2025 Sep 11;20(9):e0331920. doi: 10.1371/journal.pone.0331920 (PMC12425225; doi:10.1371/journal.pone.0331920)
Supplement: Attachment — Submitted filename: Response to Reviewers.docx [file pone.0331920.s008.docx]

We would like to thank the Academic Editor and the Reviewers for their constructive feedback and helpful suggestions. We have revised the manuscript carefully to address all comments. Below, we provide a detailed, point-by-point response. Page and line numbers refer to the revised manuscript with tracked changes.

**Requirement 1: Ensure manuscript meets PLOS ONE style requirements and file naming**
**Response:** We checked the manuscript following the PLOS ONE templates for main text and title/author/affiliation pages. File names now conform to journal guidelines:

- Response to Reviewers.docx
- Revised Manuscript with Track Changes.docx
- Manuscript.docx

**Requirement 2: Role of funders**
**Response:** We amended the funding disclosure. The revised statement now reads:

“Funding: Virginia Tech Intellectual Property LAUNCH Proof-of-Concept Grant; The School of Plant and Environmental Sciences, Virginia Tech. The funders had no role in study design, data collection and analysis, decision to publish, or preparation of the manuscript.”

This is included in the revised manuscript and cover letter.

**Requirement 3: Anonymized dataset**
**Response:** All data set are anonymized

**Requirement 4: Reviewer-recommended citations**
**Response:** No additional citations were required by the reviewers. We evaluated suggested literature and determined that no changes were needed.

**Requirement 5: References**
**Response:** We carefully reviewed the reference list. No retracted papers were found. References were updated to ensure accuracy and formatting according to PLOS ONE style.

**Reviewer #1**

**Comment R1-1:** The article is very well structured. The experiments support the proposal made in the article, clearly demonstrating the proposal of a new species of the genus *Pseudomonas*. My unique question is why in the Table 3 title describe the value as GGDC, but in the table itself, the value is labeled as dDDH?

**Response:** We thank the reviewer for highlighting this discrepancy. The inconsistency was due to terminology: the Genome-to-Genome Distance Calculator (GGDC) outputs digital DNA–DNA hybridization (dDDH) values. We have corrected the table caption and column heading for consistency. The column now reads “dDDH (%)”, and the revised caption states:

“Table 3. ANI (%) and dDDH (%) values between ‘*Candidatus* Pseudomonas auctus’ nov. sp. JDE115 and closely related sequenced strains of the genus *Pseudomonas*. Average Nucleotide Identity (ANI) and dDDH values were calculated using the Genome-to-Genome Distance Calculator (GGDC). ANI values above 95% typically indicate species-level relatedness, while dDDH values above 70% suggest the same species.”

This resolves the inconsistency.

**Reviewer #2**

**Comment R2-1:** The manuscript demonstrates strong technical support for the results and conclusions. All data (figures, tables, videos and supplementary information) are fully available. I suggest increasing the resolution of Figures 2 and 3.

**Response:** We thank the reviewer for this suggestion. Figures 2 and 3 were originally prepared as high-quality scalable vector graphics (SVG) files. During the submission process, these were converted to PDF format for review display, which may have affected the apparent resolution. The figures we submitted are high quality.

**Summary of Changes**

- Checked manuscript to PLOS ONE style and corrected file names.
- Added role of funders statement (“The funders had no role…”).
- Reviewed references for accuracy; no retracted articles included.
- Corrected Table 3 caption and column to use consistent terminology (“dDDH (%)”).
- Resolution of Figures 2 and 3 were checked and found to be suitable.

We believe these revisions fully address the comments of the reviewers and the requirements of the Academic Editor. We thank the Editor and Reviewers for their time and valuable feedback, which helped to improve the manuscript.

Sincerely,
Jonathan D. Eisenback
On behalf of all authors
